# The worldwide burden of HIV in transgender individuals: An updated systematic review and meta-analysis

**DOI:** 10.1371/journal.pone.0260063

**Published:** 2021-12-01

**Authors:** Sarah E. Stutterheim, Mart van Dijk, Haoyi Wang, Kai J. Jonas

**Affiliations:** Department of Work and Social Psychology, Maastricht University, Maastricht, The Netherlands; British Columbia Centre for Excellence in HIV/AIDS, CANADA

## Abstract

**Introduction:**

Transgender individuals are at risk for HIV. HIV risks are dynamic and there have been substantial changes in HIV prevention (e.g., pre-exposure prophylaxis [PrEP]). It is thus time to revisit HIV prevalence and burden among transgender individuals. The objective of this systematic review and meta-analysis was thus to examine worldwide prevalence and burden of HIV over the course of the epidemic among trans feminine and trans masculine individuals.

**Methods:**

We conducted an updated systematic review by searching PsycINFO, PubMed, Web of Science, and Google Scholar, for studies of any research design published in in a peer-reviewed journal in any language that reported HIV prevalence among transgender individuals published between January 2000 and January 2019. Two independent reviewers extracted the data and assessed methodological quality. We then conducted a meta-analysis, using random-effects modelling, to ascertain standardized prevalence and the relative burden of HIV carried by transgender individuals by country and year of data collection, and then by geographic region. We additionally explored the impact of sampling methods and pre-exposure prophylaxis (PrEP).

**Results:**

Based on 98 studies, overall standardized HIV prevalence over the course of the epidemic, based on weights from each country by year, was 19.9% (95% CI 14.7% - 25.1%) for trans feminine individuals (n = 48,604) and 2.56% (95% CI 0.0% - 5.9%) for trans masculine individuals (n = 6460). Overall OR for HIV infection, compared with individuals over age 15, was 66.0 (95% CI 51.4–84.8) for trans feminine individuals and 6.8 (95% CI 3.6–13.1) for trans masculine individuals. Prevalence varied by geographic region (13.5% - 29.9%) and sampling method (5.4% - 37.8%). Lastly, PrEP effects on prevalence could not be established.

**Conclusion:**

Trans feminine and trans masculine individuals are disproportionately burdened by HIV. Their unique prevention and care needs should be comprehensively addressed. Future research should further investigate the impact of sampling methods on HIV prevalence, and monitor the potential impact of PrEP.

## Introduction

Transgender individuals, defined as individuals who experience a misalignment between the sex they were assigned at birth and their gender identity or whose gender identity is incongruent with gender norms, [1, 2] are at significant risk for an HIV infection [3]. Individual level risk factors include condomless sex, particularly receptive anal sex, coinfection with other sexually transmitted infections, transactional sex, and the shared use of needles for hormone and/or silicon injections [4–8]. Individual level risk factors do not stand alone; they result from, and intersect with, other factors such as mental health difficulties, substance use, and many forms of marginalization and stigmatization that limit, among other things, educational and work opportunities, as well as legal recognition of one’s chosen gender [6, 9–14]. Given that HIV risk among transgender individuals is a dynamic phenomenon, it is important to regularly monitor and update our knowledge of HIV prevalence and burden, such that we can identify trends that can inform policy-making and interventions. Here, we present a comprehensive updated systematic review of HIV prevalence over the course of the epidemic and a meta-analyses of HIV burden among transgender individuals covering literature from 2000 until 2019.

### Previous systematic reviews and meta-analyses

Since 2008, a series of systematic reviews and meta-analyses have been published [1, 4, 5, 11, 15–17]. The first, by Herbst and colleagues, [15] investigated HIV prevalence among trans individuals in the United States, covering literature from 1988 until early 2007, and included laboratory-confirmed and self-reported prevalence. Pooled HIV prevalence based upon studies reporting laboratory-confirmed HIV status was, for trans women, 27.7%. HIV prevalence among trans women based upon self-reported HIV status was 11.8%. Among trans men, only one study reported laboratory confirmed prevalence (2%) and self-reported prevalence rates ranged from 0% to 3%. The second systematic review and meta-analysis by Operario et al. [16] set out to assess whether transgender female sex workers (FSW) experienced higher HIV infection rates than cis-gender sex workers and transgender women who do not engage in sex work, using both laboratory-confirmed and self-reported HIV prevalence rates published between 1998 and 2006. HIV prevalence was 27.3% in transgender FSW and 14.7% in trans women who did not engage in sex work. Operario et al.’s meta-analysis further showed that transgender FSW are at significantly higher risk for HIV than cis-gender sex workers and trans women who do not engage in sex work [16]. In 2013, Baral and colleagues [4] published a systematic review and meta-analysis of HIV prevalence among transgender women, covering literature from 2000 to November 2011 and using only laboratory-confirmed HIV prevalence rates. Pooled prevalence was 19.1% and the meta-analytical findings showed that, compared to all adults of reproductive age, the odds ratio for HIV infection in trans women was 49 across the 15 countries included, thus demonstrating that transgender women carry a high burden of HIV [4]. Poteat and colleagues [5] followed up on Baral et al.’s work with a systematic review, but not a meta-analysis, of HIV prevalence literature published between 2012 and 2015, looking now at both trans feminine and trans masculine populations. Prevalence rates varied substantially based on locale but Poteat et al. [5] concluded, in line with the previous reviews, that HIV prevalence was high in trans feminine populations. They also concluded that data on HIV among trans masculine individuals is still very limited. Almost simultaneously, Reisner and Murchison [1] published a global research synthesis of HIV and STI risks in adult trans men, but not trans women, using 25 studies. They found HIV prevalence rates for trans men ranging from 0% to 4.3% for laboratory-confirmed HIV status and 0% to 10% for self-reported HIV, suggesting that trans masculine individuals may also be more vulnerable to HIV than cis-gender adults. Additionally, in 2017, MacCarthy and colleagues [11] published a global systematic review of HIV and sexually transmitted infections among transgender individuals. They reported HIV prevalence rates ranging from 0% to 17.6% for self-reported HIV status and 0.6% to 34.1% for laboratory-confirmed HIV status. However, given their focus on HIV and STI co-infection, the HIV prevalence rates reported in that review were derived only from studies that also reported STI prevalence rates. The reported prevalence rates were thus based on a mere 6 studies for self-reported HIV status and 13 studies for laboratory-confirmed HIV status. Recently, Becasen et al. [17] published a systematic review and meta-analysis of HIV prevalence among transgender individuals in the United States only using literature published in the United States between 2006 and May 2017. They established that laboratory-confirmed HIV prevalence was 14.1% for trans women and 3.2% for trans men; self-reported prevalence was 16.1% and 1.2% for trans women and trans men, respectively.

### Current concerns

Overall, the various systematic reviews and meta-analyses demonstrate that transgender individuals, particularly trans feminine individuals, are disproportionately burdened with HIV but none of the more recent systematic reviews have comprehensively updated Baral et al.’s worldwide systematic review and meta-analysis with both transfeminine and transmasculine individuals. Furthermore, from a methodological perspective, more fine-grained analyses (e.g. by country and year of data collection) are being called for, rather than only pooled analyses by country or region, as has been the methodological approach in previous meta-analyses. Additionally, critique about reported prevalence rates has been levied, with the claim that many studies have relied on convenience samples of, often, transgender women who engage in sex work, which may inflate prevalence rates [18, 19]. Also, previous meta-analyses have not differentiated between various sampling strategies and this may impact meta-analytical findings. Further, there have been substantial and fundamental changes in HIV prevention in recent years. One is the emergence of pre-exposure prophylaxis (PrEP) as a powerful tool for HIV prevention for at risk groups like transgender individuals [5, 8, 20–23]. With these considerations in mind, we feel it is time to revisit worldwide HIV prevalence and burden among transgender individuals. We therefore systematically reviewed literature published between 2000 and 2019 on HIV prevalence among transgender individuals and then conducted a meta-analysis 1) to establish prevalence rates for both trans feminine and trans masculine individuals; and 2) to compare the burden of HIV infection among transgender individuals to individuals over 15 years of age in the countries and regions from which samples were derived, taking year of data collection into account. We then explored the possible impact of sampling methods and of PrEP on prevalence rates and the burden of HIV infection.

## Methods

### Search strategies and eligibility

We searched, in November 2017 and again in January 2019, PsycINFO, PubMed, Web of Science, and Google Scholar®, for studies in all languages published between January 1st, 2000 and January 28^th^, 2019. We selected this timeframe in order to gain a complete, comprehensive, and nuanced understanding of worldwide prevalence over and burden of HIV among transgender individuals. We also reviewed the studies included in Baral et al. [4] and in Poteat et al. [5] to ensure that they were covered in our analysis as well. We explicitly overlapped the timeframe in our meta-analysis with those of previous meta-analyses in order to generate comprehensive and robust meta-analytical findings. It also allowed us to explore the impact of applying more refined methodology (standardized vs. pooled prevalence rates) in the meta-analysis, and compare findings delivered by the different meta-analytical approaches. Articles and citations were downloaded and managed in the reference software Mendeley®.

We searched for articles on (the treatment of) HIV and transgender individuals using the following search terms: HIV OR AIDS OR “PrEP” OR “Pre-Exposure Prophylaxis” OR “TasP” OR “treatment as prevention” AND *transgender* OR “MTF” or “male to female transgender” OR “FTM” OR “female to male transgender” OR *transsexual* OR “travesty” OR “cross dresser” OR “koti” OR “hijra” OR “mahuvahine” OR “mahu” OR “waria” OR “katoey” OR “bantut” OR “nadleehi” OR “berdache” OR “xanith”. These terms are in line with the terms previously used by Baral et al. [4].

Studies of any research design published in peer-reviewed journals that reported laboratory- confirmed prevalence of HIV among transgender individuals were included. When prevalence rates were pooled across trans feminine and trans masculine individuals or when prevalence was pooled across trans feminine individuals and men who have sex with men (MSM), we contacted the authors and requested separate prevalence rates for the populations included.

### Study selection and data extraction

Titles and abstracts were screened by two independent reviewers and articles that clearly did not include HIV prevalence data were excluded, as were duplicates. All articles that met the inclusion criteria and articles that needed further review to ascertain whether they met the criteria were subsequently downloaded. When one reviewer deemed the title and/or abstract potentially relevant and the other did not, the full-text for that article was nonetheless downloaded. Subsequently, the full texts were reviewed. When studies reported duplicate data, the study with the smallest sample size was excluded. If sample sizes were identical, the later publication was excluded. Any conflicts over study inclusion were resolved by project leads (KJ and SS) in conjunction with the researchers running the meta-analysis (MvD and HW). The PRISMA reporting checklist was used to guide the reporting of this study. No protocol was registered for this review.

Data were extracted by two trained coders using a standardized extraction form that included details about sample size, sampling method, sample description, recruitment location, time period of study, age range, transgender type (trans masculine/trans feminine/both), HIV measure (self-reported/laboratory testing), and HIV prevalence or incidence.

### Methodological quality assessment

Given the lack of consensus on fitting quality assessment tools for epidemiological studies, [24] we developed criteria specifically for this systematic review and meta-analysis. In doing so, we used and adapted appropriate criteria from the JBI Critical Appraisal Checklist for Studies Reporting Prevalence Data [25]. Studies were deemed of sufficient quality if: 1) biological testing (rather than self-reported HIV status) was used to establish HIV diagnoses, as was done in Baral et al. [4]; 2) study participants were described in sufficient detail, meaning that prevalence was reported, or subsequently obtained directly from the authors, specifically for trans feminine and/or trans masculine individuals (rather than transgender individuals as a whole group); 3) if the study setting/location was sufficiently detailed; 4) if the data collection timeframe was reported; 5) if prevalence or frequency of HIV diagnosis within the total sample were reported; and 6) if sample size was at least 40 for trans feminine individuals. We did not apply a minimum sample size for studies reporting prevalence among trans masculine individuals as the majority of studies had small sample sizes, and a minimum sample size would have led to the exclusion of most studies reporting HIV prevalence among trans masculine individuals. Additionally, we did not exclude studies based on sampling method as investigating the impact of sampling methods was one of the objectives of this meta-analysis.

### Data analyses

First, we used analogous methodology to prior meta-analyses [4, 26, 27]. We grouped studies by country, weighted by sample size. We calculated pooled HIV prevalence and 95% confidence intervals (CIs) per country. We did this separately for trans feminine and trans masculine samples. In line with previous meta-analyses, we then calculated odds ratios per country by dividing the HIV prevalence among transgender individuals (numerator) by the HIV prevalence rate among individuals over 15 years of age in the general population in the country from which the sample was derived (denominator), as reported by the 2017 UNAIDS reports (where prevalence estimates for adults are from 15 years of age onward) [28] and estimations of adult population size from the US Census Bureau International Division [29]. These results are reported in S1 Appendix.

Then, to achieve a more refined methodological analysis, we standardized rather than pooled prevalence rates, and ran the meta-analysis again, this time matching country-level prevalence rates to year(s) of data collection for the included studies. When data were collected over multiple years in the original studies, the median year of the year-span was chosen for the country by year analysis. If HIV prevalence in the sample was 0, we calculated confidence intervals using the Wilson interval [30]. Then, we grouped countries by geographic region (Africa, Latin America, Asia, and Global North) and calculated, per geographic region, the standardized HIV prevalence among trans feminine individuals as well as odds ratios based on weights from each country-year.

Subsequently, given recent discussions about the impact of sampling methods on findings pertaining to HIV prevalence among trans feminine individuals, [18] we grouped studies by sampling method, and calculated standardized HIV prevalence by sampling method. We delineated ten sampling methods, namely cluster sampling, convenience sampling, purposive sampling, respondent driven sampling, snowball sampling, sampling from database health plan, as well as sampling via STI clinic, via hospital, via NGO, and via surveillance. Overlap in categories may exist as some studies used multiple sampling methods. In such cases, we categorized the study under its primary sampling method.

Lastly, we explored possible effects of the introduction of PrEP on HIV prevalence among trans feminine individuals. We focused on US studies only as PrEP has been available in the US since 2012, which is longer than in any other country. We conducted subgroup analyses, with data being collected either prior to the introduction of PrEP (1997–2011), or after the introduction of PrEP (2012–2017).

The meta-analysis was conducted with the statistical software R [31] using the metafor package [32]. We used a random-effects model and the DerSimonian-Laird method to estimate the model. The DerSimonian-Laird *Q* test and *I*^*2*^ values were used to assess heterogeneity, with low, moderate, and high heterogeneity corresponding to *I*^*2*^ values of 25%, 50%, and 75%.[33] We investigated publication bias by inspecting funnel plots [34].

## Results

**Fig 1:**
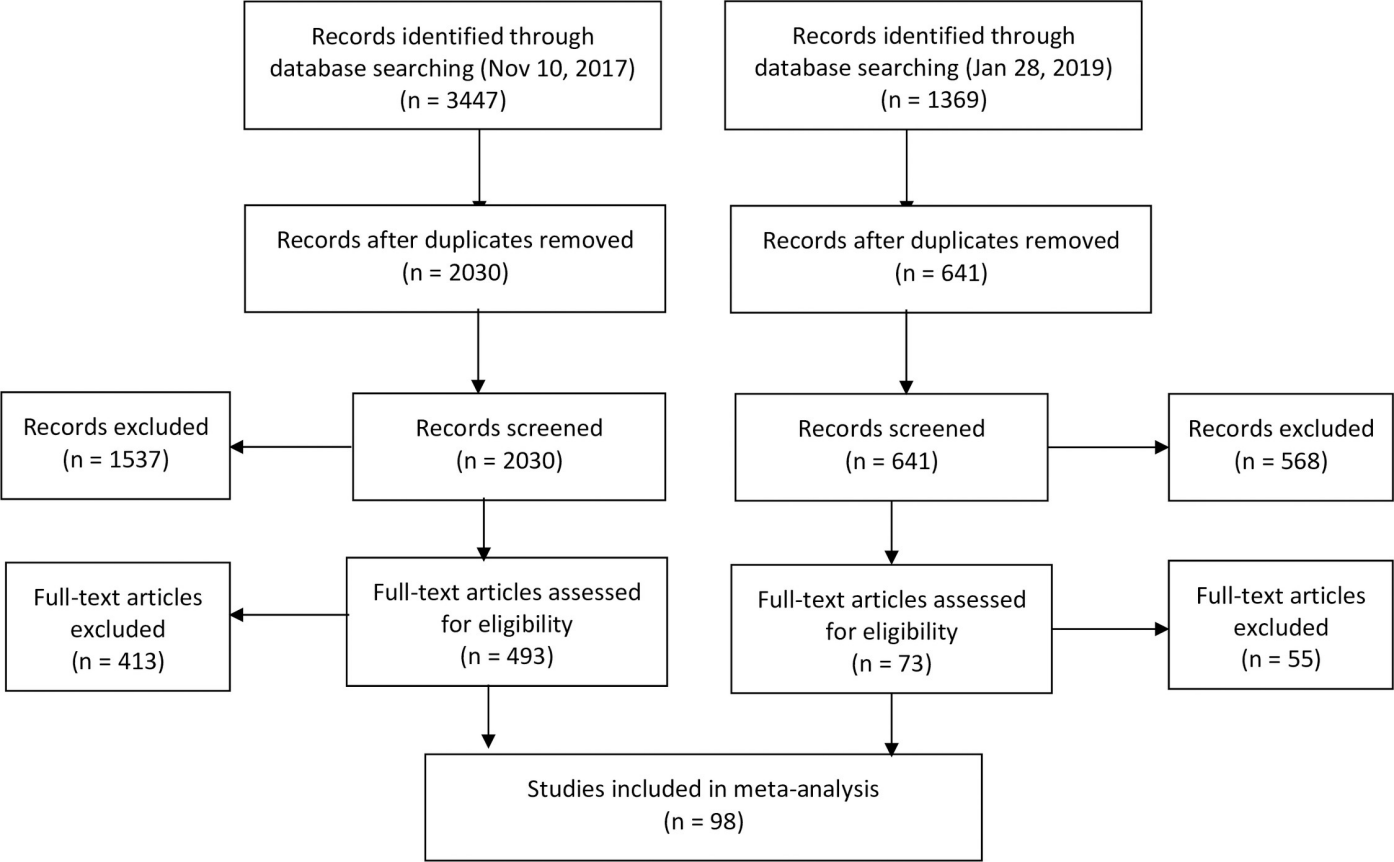
PRISMA flow chart describing the study selection process.

The study selection process is presented in Fig 1. We included 98 studies from a total of 34 countries, of which 78 studies described HIV prevalence in trans feminine individuals, 4 described prevalence in trans masculine individuals, and 16 described both. In total, we included 48,604 trans feminine individuals from 34 countries and 6460 trans masculine individuals from 5 countries. The included studies and relevant characteristics of those studies are reflected in Table 1.

**Table 1:** Studies included in review and meta-analysis.

Authors	Year of publication	Year of data collection	Transgender sample	HIV prevalence (%)	HIV frequency (n)	Sample size	Country	Geographic region	Sampling method
Aguayo, Munoz, & Aguilar [35]	2013	2011	TF	27.00%	64	237	Paraguay	Latin America	Cluster sampling
Akhtar, Badshah, Akhtar, et al. [36]	2012	2009–2010	TF (hijras)	21.60%	66	306	Pakistan	Asia	Respondent driven sampling
Altaf [37]	2009	2006–2007	TF (hijras)	4.70%	38	810	Pakistan	Asia	Surveillance
Altaf, Zahidie, & Agha [38]	2012	2008	TF (hijras)	6.40%	75	1181	Pakistan	Asia	Surveillance
Baqi, Shah, Baig et al. [39]	2006	1998	TF (hijras)	0.00%	0	208	Pakistan	Asia	Respondent driven sampling
Barrington, Weijnert, & Guardado et al. [40]	2012	2008	TF	19.00%	13	67	El Salvador	Latin America	Respondent driven sampling
Bastos, Bastos, Coutinho et al. [41]	2018	2016–2017	TF	29.62%	843	2846	Brazil	Latin America	Respondent driven sampling
Bellhouse, Walker, Fairley et al. [42]	2016	2011–2014	TM	3.57%	1	28	Australia	Global North	STI clinic visit
			TF	10.39%	8	77			
Brahmam, Kodavallaa, Rajkumar et al. [43]	2008	2006–2007	TF (hijras)	18.10%	104	575	India	Asia	Cluster sampling
Carballo-Dieguez, Balan, Dolezal et al. [44]	2012	2005–2006	TF	13.00%	12	84	Brazil	Latin America	Respondent driven sampling
Castel, Magnus, Peterson et al. [45]	2012	2006	TF & TM	10.59%	9	85	US	Global North	STI clinic visit
Castillo, Konda, Leon et al. [46]	2015	2008–2009	TF	16.82%	35	208	Peru	Latin America	Snowball
Chariyalersak, Kosachunhanan, Saokhieo et al. [47]	2011	2008–2009	TF	9.30%	13	140	Thailand	Asia	STI clinic visit
Chen, McFarland, Tompson et al. [48]	2011	2009	TM	0.00%	0	59	US	Global North	STI clinic visit
Chhim, Ngin, Chhoun et al. [49]	2017	2015–2016	TF	5.90%	81	1375	Cambodia	Asia	Respondent driven sampling
Clements-Noelle, Wilkenson, Kitano et al. [50]	2001	1997	TM	2.00%	2	123	US	Global North	Respondent driven sampling
			TF	35.00%	137	392			
Colby, Nguyen, Le et al. [51]	2016	2015	TF	18.00%	37	205	Vietnam	Asia	Snowball
Costa, Fontanari, Jacinto et al. [52]	2015	1998–2014	TM	25.00%	0	51	Brazil	Latin America	Hospital
			TF	25.00%	71	284			
Dasarathan & Kalaivani [53]	2017	2011–2014	TF	13.40%	11	82	India	Asia	STI clinic visit
Diez, Bleda, Varela et al. [54]	2014	2000–2009	TF	24.50%	129	529	Spain	Global North	STI clinic visit
Dos Ramos Farias, Garcia, Reynaga et al. [55]	2011	2006–2009	TF	34.10%	93	273	Argentina	Latin America	Respondent driven sampling
Fernandes, Zanini, Rezende et al. [56]	2015	2011–2013	TF	24.34%	37	152	Brazil	Latin America	Cluster sampling
Fernandez-Balbuena, Belza, Urdaneta et al. [57]	2015	2008–2012	TF & TM	45.54%	46	101	Spain	Global North	NGO
Fernandez-Lopez, Reyes-Uruena, Agusti et al. [58]	2018	2014–2016	TF	8.83%	40	453	Spain	Global North	STI clinic visit
Grandi, Goihman, Ueda et al. [59]	2000	1992–1998	TF	40.00%	174	434	Brazil	Latin America	Respondent driven sampling
Green, Hoenigl, Morris et al. [60]	2015	2008–2014	TM	3.00%	1	30	US	Global North	STI clinic visit
			TF	2.00%	3	151			
Grinsztejn, Jalil, Monteiro et al. [61]	2017	2015–2016	TF	31.20%/ 24.20%	141	345	Brazil	Latin America	Respondent driven sampling
Guadamuz, Wimonsate, Varangrat et al. [62]	2011	2005	TF	14.00%	64	474	Thailand	Asia	Convenience sampling
Gutierrez, Tajada, Alvarez et al. [63]	2004	1998–2003	TF	23.00%	14	60	Spain	Global North	Convenience sampling
Guy, Mustikawati, Wijaksono et al. [64]	2011	2006–2008	TF & TM	31.60%	151	477	Indonesia	Asia	STI clinic visit
Habarta, Wang, Mulatu et al. [65]	2015	2009–2011	TM	0.51%	12	2364	US	Global North	STI clinic visit
			TF	2.70%	355	13154			
Hadikusumo, Utsumi, Amin et al. [66]	2016	2012	TF	16.00%	16	100	Indonesia	Asia	STI clinic visit
Hakim, Coy, Patnaik et al. [67]	2018	2014–2015	TF	22.42%	37	165	Mali	Africa	Respondent driven sampling
Hawkes, Collumbien, Platt et al. [68]	2009	2007	TF (khusra)	2.00%	6	269	Pakistan	Asia	Respondent driven sampling
Hiransuthikul, Pattanachaiwit, Teeratakulpisarn et al. [69]	2018	2012–2013	TF	4.26%	2	47	Thailand	Asia	STI clinic visit
Januraga, Wulandari, Muliawan et al. [70]	2013	2009–2010	TF (waria)	36.87%	80	217	Indonesia	Asia	Respondent driven sampling
Jin, Restar, Biello et al. [71]	2019	2012–2015	TF	24.71%	65	263	US	Global North	Convenience sampling
Kaplan, McGowan, & Wagner [72]	2016	2012	TF	10.00%	4	40	Lebanon	Asia	Respondent driven sampling
Kellogg, Clements-Nolle, Dilley et al. [73]	2001	1997–2000	TF	15.00%	37	238	US	Global North	STI clinic visit
Keshinro, Crowell, Nowak et al. [74]	2016	2013–2016	TF	71.43%	75	105	Nigeria	Africa	Respondent driven sampling
Khan, Rehan, Qayyum et al. [75]	2008	2004	TF (hijras)	1.00%	5	409	Pakistan	Asia	Cluster sampling
Kojima, Park, Konda et al. [76]	2017	2013–2014	TF	30.10% / 27.60%	30	89	Peru	Latin America	STI clinic visit
Leinung, Urizar, Patel et al. [77]	2013	prior 2003	TM	0.00%	0	50	US	Global North	Hospital
			TF	8.33%	16	192			
Lipsitz, Segura, Castro et al. [78]	2014	2007–2009	TF	30.00%	64	214	Peru	Latin America	STI clinic visit
Lobato, Koff, Schestatsky et al. [79]	2008	1998–2005	TM	0.00%	0	16	Brazil	Latin America	Hospital
			TF	19.67%	24	122			
Logie, Lacombe-Duncan, Wang et al. [80]	2016	2015	TF	25.20%	26	103	Jamaica	Latin America	Respondent driven sampling
Long, Montano, Cabello et al. [81]	2017	2013–2015	TF	19.68%	61	310	Peru	Latin America	STI clinic visit
Luzzati, Zatta, Pavan et al. [82]	2016	2000–2014	TM	0.00%	0	20	Italy	Global North	Hospital
			TF	12.10%	21	173			
Manieri, Castellano, Crespi et al. [83]	2014	2005–2011	TM	0.00%	0	27	Italy	Global North	Hospital
			TF	5.36%	3	56			
McFarland, Wilson, Raymond et al. [84]	2017	2014	TM	0.00%	0	122	US	Global North	Convenience sampling
Mimiaga, Hughto, Biello et al. [85]	2019	2012–2015	TF	20.60%	48	233	US	Global North	Convenience sampling
Murrill, Liu, Guilin et al. [86]	2008	2004	TF & TM	13.00%	9	92	US	Global North	Convenience sampling
Nemoto, Bödeker, Iwamoto et al. [87]	2014	2000–2001	TF	29.93%	161	538	US	Global North	Purposive sampling
Nguyen, Nguyen, Le et al. [88]	2008	2004	TF ("male transvestites" "bong lo")	7.00%	5	75	Vietnam	Asia	Convenience sampling
Nuttbrock, Bockting, Rosenblum et al. [89]	2013	2004–2007	TF	2.80%	9	230	US	Global North	Convenience sampling
Nuttbrock, Hwahng, Bockting et al. [90]	2009	earlier than 2009	TF	35.98%	186	517	US	Global North	Convenience sampling
Ongwandee, Lertpiriyasuwat, Khawcharoenporn et al. [91]	2018	2015–2016	TF	900.00%	39	435	Thailand	Asia	STI clinic visit
Pando, Gomez-Carrillo, Vignoles et al. [92]	2011	2006–2008	TF	34.00%	38	112	Argentina	Latin America	NGO
Patrascioiu, Lopez, Porta et al. [93]	2013	2006–2010	TM	2.20%	2	92	Spain	Global North	Convenience sampling
			TF	12.60%	18	142			
Peitzmeier, Reisner, Harigopal et al. [94]	2014	2006–2012	TM	0.86%	2	233	US	Global North	Hospital
Pell, Prone, Vlahakis et al. [95]	2011	2004	TM	0.00%	0	17	Australia	Global North	STI clinic visit
			TF	4.26%	6	141			
Pisani, Girault, Gultom et al. [96]	2004	2002	TF (waria)	22.00%	53	241	Indonesia	Asia	Cluster sampling
Pitasi, Oraka, Clark et al. [97]	2019	2010–2013	TM	8.30%	10	120	US	Global North	STI clinic visit
			TF	14.20%	72	506			
Pizzicato, Vagenas, Gonzales et al. [98]	2017	2011	TF	14.59%	104	713	Peru	Latin America	Respondent driven sampling
Poteat, Ackerman, Diouf et al. [99]	2017	2011–2016	TF	2.78%	3	108	Burkina Faso	Africa	Respondent driven sampling
			TF	25.50%	76	298	Côte d’Ivoire		
			TF	59.15%	42	71	Lesotho		
			TF	16.00%	12	75	Malawi		
			TF	37.19%	74	199	Senegal		
			TF	14.17%	17	120	Swaziland		
			TF	17.65%	9	51	Togo		
Poteat, German, & Flynn [100]	2016	2004–2005	TF	43.00%	21	49	US	Global North	Surveillance
Prabawanti, Bollen, Palupy et al. [101]	2011	2007	TF (waria)	24.40%	183	748	Indonesia	Asia	Cluster sampling
Quinn, Nash, Hunkeler et al. [102]	2017	2006–2014	TM	0.31%	9	2892	US	Global North	Database health plan
			TF	5.35%	186	3475			
Rana, Reza, Alam et al. [103]	2016	2012	TF (hijras)	0.80%	7	889	Bangladesh	Asia	STI clinic visit
Raymond, Wilson, Packer et al. [104]	2019	2010	TF	39.17%	123	314	US	Global North	Respondent driven sampling
		2013	TF	36.05%	84	233			
		2016	TF	38.68%	123	318			
Reback, Lombardi, Simon et al. [105]	2005	1998–1999	TF	22.10%	54	244	US	Global North	STI clinic visit
Reisner, White, Mayer et al. [106]	2014	2007	TM	4.35%	1	23	US	Global North	STI clinic visit
Reisner, Vetters, White et al. [107]	2015	2001–2010	TF	7.93%	5	63	US	Global North	STI clinic visit
			TM	2.40%	2	82			
Rich, Scott, Johnston, et al. [108]	2017	2012–2014	TM	0.00%	0	11	Canada	Global North	Respondent driven sampling
Rowe, Santos, McFarland et al. [109]	2015	2012–2013	TF	4.00%	13	292	US	Global North	Snowball
Russi, Serra, Vinoles et al. [110]	2003	1999	TF ("male transvestites")	21.50%	49	200	Uruguay	Latin America	Convenience sampling
Sahastrabuddhe, Gupta, Stuart et al. [111]	2012	1993–2002	TF (hijras)	45.20%	38	84	India	Asia	STI clinic visit
Salas-Espinoza, Menchaca-Diaz, Patterson et al. [112]	2017	2012	TF	22.00%	22	100	Mexico	Latin America	Cluster sampling
Saravanamurthy, Rajendran, Ramakrishnan et al. [113]	2008	2007	TF	17.50%	23	125	India	Asia	Respondent driven sampling
Schulden, Song, Barros et al. [114]	2008	2005–2006	TM	0.00%	0	42	US	Global North	Convenience sampling
			TF	12.00%	67	559			
Seekaew, Pengnonyang, Jantarapakde et al. [115]	2018	2015–2016	TF	8.80%	69	786	Thailand	Asia	Respondent driven sampling
Shan, Yu, Yang et al. [116]	2018	2016	TF	7.60%	38	498	China	Asia	Snowball
Shaw, Lorway, Bhattacharjee et al. [117]	2016	2011	TF (kothi & hijras)	15.30%	27	176	India	Asia	Cluster sampling
Shaw, Emmanuel, Adrien et al. [118]	2011	2005–2006	TF (hijras)	1.00%	10	1162	Pakistan	Asia	Cluster sampling
Sherman, Park, Galai et al. [119]	2019	2016–2017	TF	40.30%	25	62	US	Global North	Convenience sampling
Shinde, Setia, Row-Kavi et al. [120]	2009	earlier than 2009	TF	41.00%	21	51	India	Asia	STI clinic visit
Silva-Santisteban, Raymond, Salazar et al. [121]	2012	2009	TF	30.00%	130	420	Peru	Latin America	Respondent driven sampling
Sotelo & Claudia [122]	2011	2009	TF	34.00%	152	441	Argentina	Latin America	Unknown
Stephens, Bernstein, Philip et al. [123]	2011	2006–2009	TM	2.90%	7	69	US	Global North	STI clinic visit
			TF	11.21%	25	223			
Subramanian, Ramakrishnan, Aridoss et al. [124]	2013	2005–2009	TF	12.00%	48	404	India	Asia	Cluster sampling
Toibaro, Ebensrtejin, Parlante et al. [125]	2009	2002–2006	TF	27.60%	29	105	Argentina	Latin America	STI clinic visit
Van Veen, Götz, van Leeuwen et al. [126]	2010	2002–2005	TF	19.00%	13	69	Netherlands	Global North	Cluster sampling
Waheed, Satti, Arshad et al. [127]	2017	2015–2016	TF	16.40%	22	134	Pakistan	Asia	Convenience sampling
Wasantioopapokakorn, Manopaiboon, Phoorisri et al. [128]	2018	2011–2016	TF	11.85%	82	692	Thailand	Asia	Convenience sampling
Weissman, Ngak, Srean et al. [129]	2016	2012	TF	4.00%	37	891	Cambodia	Asia	Respondent driven sampling
World Health Organization [130]	2016	2015–2016	TF	4.00%	11	299	Philippines	Asia	Surveillance
Wickersham, Gibson, Bazazi et al. [131]	2017	2014	TF	12.00%	24	193	Malaysia	Asia	Respondent driven sampling
Zaccarelli, Spizzichino, Venezia et al. [132]	2004	1993–2003	TF	31.50%	149	473	Italy	Global North	STI clinic visit
Zea, Reisen, del Rio-Gonzalez et al. [133]	2015	2011	TF	13.79%	8	58	Colombia	Latin America	Respondent driven sampling

TF = trans feminine; TM = trans masculine.

**Fig 2:**
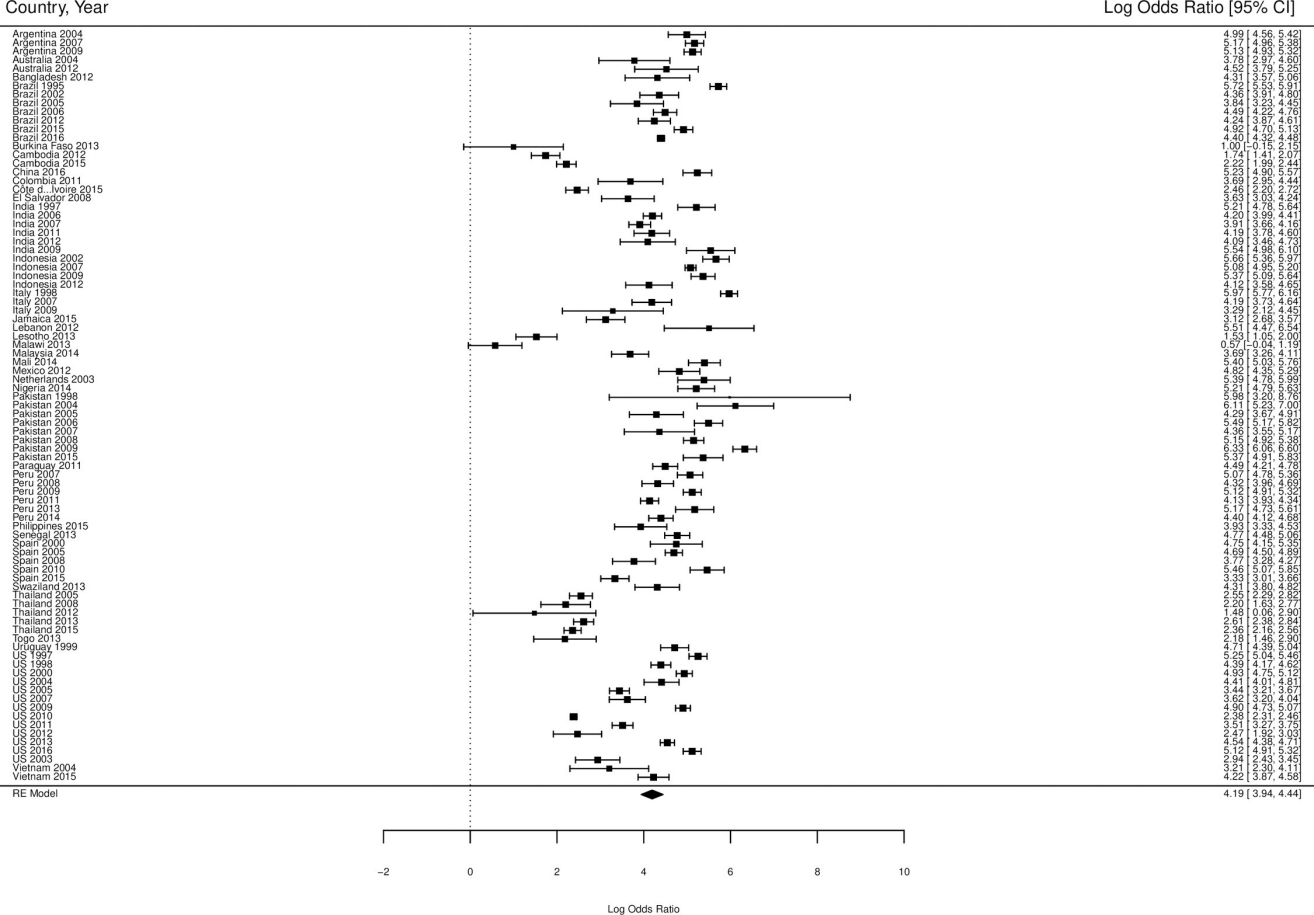
Forest plot of HIV prevalence in trans feminine individuals compared to all adults (age 15+). The scale on the x-axis is log odds ratio. The percentages indicate the weight of each country by year within the meta-analysis. The numbers in the right column are the log odds ratios including their confidence intervals. We converted these log odds ratios into odds ratios, as described in *Table 2*.

**Fig 3:**
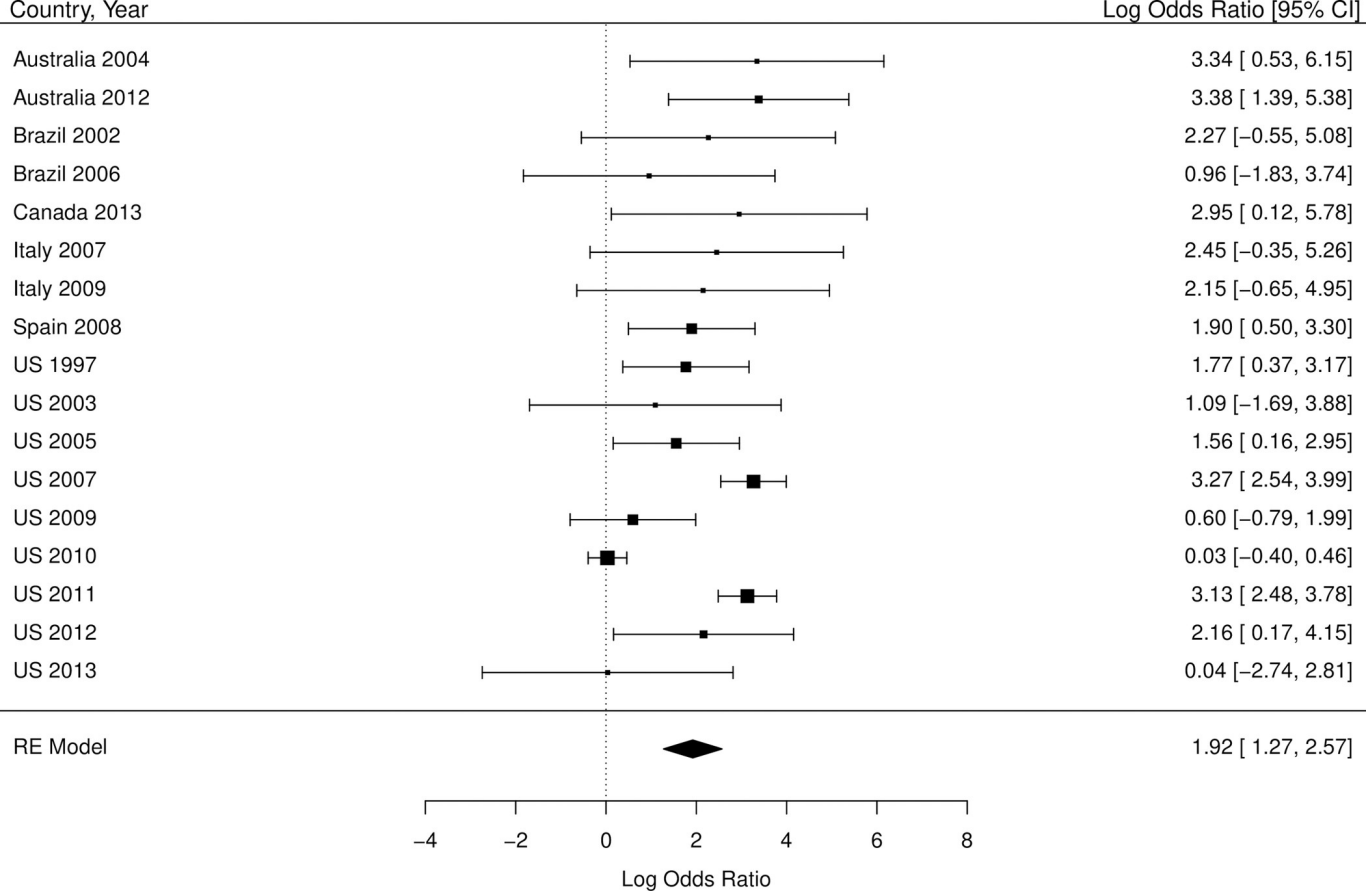
Forest plot of HIV prevalence in trans masculine individuals compared to all adults (age 15+). The scale on the x-axis is log odds ratio. The percentages indicate the weight of each country by year within the meta-analysis. The numbers in the right column are the log odds ratios including their confidence intervals. We converted these log odds ratios into odds ratios, as described in *Table 3*.

The overall standardized HIV prevalence over the course of the epidemic, based on weights from each country by year, was 19.9% (95% CI 14.7% - 25.1% Table 2) for trans feminine individuals and 2.56% (95% CI 0.0% - 5.9%; Table 3) for trans masculine individuals. The overall OR for HIV infection, compared with individuals over 15 years of age, was 66.0 (95% CI 51.4–84.8; Table 2 and Fig 2) for trans feminine individuals and 6.8 (95% CI 3.6–13.1, Table 3 and Fig 3) for trans masculine individuals. Tables 2 and 3 also show the overall standardized prevalence rates and overall odds ratios per country by year for trans feminine individuals and trans masculine individuals, respectively.

**Table 2:** Meta-analysis of HIV prevalence in trans feminine individuals compared to all adults (age 15+).

Country	Year of data collection	Number of samples	Sample size	Frequency of HIV among TF in the samples	Prevalence (95% CI)*	Odds Ratio (95%CI)	HIV prevalence in adults (95% CI)
Argentina	2004	1	105	29	27.6 (19.1–36.2)	147.2 (96.0–225.9)	0.258 (0.257–0.260)
Argentina	2007	2	385	131	34 (29.3–38.8)	176 (142.6–217.4)	0.292 (0.290–0.294)
Argentina	2009	1	441	152	34.5 (30–38.9)	168.3 (138.3–204.8)	0.312 (0.310–0.314)
Australia	2004	1	141	6	4.3 (0.9–7.6)	44 (19.4–99.7)	0.101 (0.099–0.102)
Australia	2012	1	77	8	10.4 (3.6–17.2)	92.1 (44.3–191.5)	0.126 (0.124–0.127)
Bangladesh	2012	1	889	7	0.8 (0.2–1.4)	74.7 (35.5–157.3)	0.011 (0.010–0.011)
Brazil	1995	1	434	174	40.1 (35.5–44.7)	304.8 (251.6–369.3)	0.219 (0.218–0.220)
Brazil	2002	1	122	24	19.7 (12.6–26.7)	78 (49.9–121.9)	0.313 (0.312–0.314)
Brazil	2005	1	84	12	14.3 (6.8–21.8)	46.7 (25.3–86.0)	0.356 (0.355–0.357)
Brazil	2006	1	284	71	25.0 (20.0–30.0)	89.3 (68.2–116.8)	0.372 (0.371–0.373)
Brazil	2012	1	152	37	24.3 (17.5–31.2)	69.7 (48.1–100.9)	0.460 (0.459–0.461)
Brazil	2015	1	345	141	40.9 (35.7–46.1)	136.4 (110.1–169.1)	0.504 (0.503–0.505)
Brazil	2016	1	2846	843	29.6 (27.9–31.3)	81.1 (74.9–87.9)	0.516 (0.515–0.517)
Burkina Faso	2013	1	108	3	2.8 (-0.3–5.9)	2.7 (0.9–8.5)	1.043 (1.036–1.049)
Cambodia	2012	1	891	37	4.2 (2.8–5.5)	5.7 (4.1–7.9)	0.756 (0.751–0.762)
Cambodia	2015	1	1375	81	5.9 (4.6–7.1)	9.2 (7.3–11.5)	0.678 (0.673–0.683)
China	2016	1	498	38	7.6 (5.3–10)	187.7 (134.8–261.3)	0.044 (0.044–0.044)
Colombia	2011	1	58	8	13.8 (4.9–22.7)	40.2 (19.1–84.8)	0.397 (0.394–0.399)
Côte d’Ivoire	2015	1	298	76	25.5 (20.6–30.5)	11.7 (9.1–15.2)	2.832 (2.823–2.841)
El Salvador	2008	1	67	13	19.4 (9.9–28.9)	37.9 (20.7–69.4)	0.631 (0.623–0.639)
India	1997	1	84	38	45.2 (34.6–55.9)	183.8 (119.6–282.4)	0.447 (0.447–0.448)
India	2006	1	575	104	18.1 (14.9–21.2)	66.7 (53.9–82.5)	0.330 (0.330–0.330)
India	2007	2	529	71	13.4 (10.5–16.3)	49.8 (38.8–63.9)	0.310 (0.310–0.311)
India	2011	1	176	27	15.3 (10.0–20.7)	65.7 (43.6–99.0)	0.275 (0.275–0.275)
India	2012	1	82	11	13.4 (6.0–20.8)	59.9 (31.7–113.0)	0.258 (0.258–0.258)
India	2009	1	51	21	41.2 (27.7–54.7)	255.3 (146.1–445.8)	0.273 (0.273–0.274)
Indonesia	2002	1	241	53	22 (16.8–27.2)	288.5 (212.7–391.4)	0.098 (0.097–0.098)
Indonesia	2007	2	1225	334	27.3 (24.8–29.8)	160.4 (141.5–181.9)	0.233 (0.232–0.234)
Indonesia	2009	1	217	80	36.9 (30.4–43.3)	214.1 (162.5–282.1)	0.272 (0.271–0.273)
Indonesia	2012	1	100	16	16.0 (8.8–23.2)	61.5 (36.0–105.0)	0.309 (0.308–0.310)
Italy	1998	1	473	149	31.5 (27.3–35.7)	390.4 (321.5–474.1)	0.118 (0.117–0.119)
Italy	2007	1	173	21	12.1 (7.3–17)	65.7 (41.7–103.8)	0.210 (0.208–0.211)
Italy	2009	1	56	3	5.4 (-0.5–11.3)	26.8 (8.4–85.6)	0.211 (0.210–0.212)
Jamaica	2015	1	103	26	25.2 (16.9–33.6)	22.7 (14.5–35.4)	1.468 (1.451–1.484)
Lebanon	2012	1	40	4	10.0 (0.7–19.3)	246.1 (87.5–692.4)	0.045 (0.043–0.047)
Lesotho	2013	1	71	42	59.2 (47.7–70.6)	4.6 (2.9–7.4)	23.950 (23.876–24.023)
Malawi	2013	1	75	12	16.0 (7.7–24.3)	1.8 (1.0–3.3)	9.683 (9.664–9.703)
Malaysia	2014	1	193	24	12.4 (7.8–17.1)	39.9 (26.0–61.1)	0.355 (0.352–0.357)
Mali	2014	1	165	37	22.4 (16.1–28.8)	220.9 (153.1–318.6)	0.131 (0.128–0.133)
Mexico	2012	1	100	22	22 (13.9–30.1)	123.9 (77.2–198.9)	0.227 (0.226–0.228)
Netherlands	2003	1	69	13	18.8 (9.6–28.1)	218.8 (119.7–400.2)	0.106 (0.104–0.108)
Nigeria	2014	1	105	75	71.4 (62.8–80.1)	183 (119.8–279.4)	1.348 (1.346–1.350)
Pakistan	1998	1	208	0	0 (0.0–0.0)	395.9 (24.6–6359.6)	0.001 (0.001–0.001)
Pakistan	2004	1	409	5	1.2 (0.2–2.3)	451.5 (186.8–1091.6)	0.003 (0.003–0.003)
Pakistan	2005	1	1162	10	0.9 (0.3–1.4)	73.0 (39.2–136.1)	0.012 (0.012–0.012)
Pakistan	2006	1	810	38	4.7 (3.2–6.1)	243.0 (175.4–336.6)	0.020 (0.020–0.021)
Pakistan	2007	1	269	6	2.2 (0.5–4)	78.4 (34.9–176.1)	0.029 (0.029–0.029)
Pakistan	2008	1	1181	75	6.4 (5–7.7)	172.4 (136.4–217.9)	0.039 (0.039–0.040)
Pakistan	2009	1	306	66	21.6 (17–26.2)	560.5 (426.8–736.1)	0.049 (0.049–0.049)
Pakistan	2015	1	134	22	16.4 (10.1–22.7)	214.8 (136.0–339.2)	0.091 (0.091–0.092)
Paraguay	2011	1	237	64	27 (21.4–32.7)	89.5 (67.2–119.3)	0.411 (0.406–0.417)
Peru	2007	1	214	64	29.9 (23.8–36)	158.9 (118.5–212.9)	0.268 (0.266–0.270)
Peru	2008	1	208	35	16.8 (11.7–21.9)	75.3 (52.4–108.3)	0.268 (0.266–0.270)
Peru	2009	1	420	130	31 (26.5–35.4)	166.9 (135.7–205.3)	0.268 (0.266–0.270)
Peru	2011	1	713	104	14.6 (12–17.2)	62.5 (50.7–76.9)	0.273 (0.270–0.275)
Peru	2013	1	89	30	33.7 (23.9–43.5)	176.6 (113.8–274.1)	0.287 (0.285–0.289)
Peru	2014	1	310	61	19.7 (15.3–24.1)	81.1 (61.3–107.4)	0.301 (0.299–0.303)
Philippines	2015	1	299	11	3.7 (1.5–5.8)	50.9 (27.9–92.9)	0.075 (0.074–0.076)
Senegal	2013	1	199	74	37.2 (30.5–43.9)	118.1 (88.6–157.4)	0.499 (0.494–0.504)
Spain	2000	1	60	14	23.3 (12.6–34)	115.6 (63.5–210.2)	0.263 (0.261–0.264)
Spain	2005	1	529	129	24.4 (20.7–28)	109.3 (89.6–133.3)	0.294 (0.293–0.296)
Spain	2008	1	142	18	12.7 (7.2–18.1)	43.6 (26.6–71.5)	0.332 (0.330–0.334)
Spain	2010	1	101	46	45.5 (35.8–55.3)	235.3 (159.1–348.1)	0.354 (0.352–0.356)
Spain	2015	1	453	40	8.8 (6.2–11.4)	28.1 (20.3–38.8)	0.344 (0.342–0.346)
Swaziland	2013	1	120	17	14.2 (7.9–20.4)	74.5 (44.6–124.4)	0.221 (0.218–0.225)
Thailand	2005	1	474	64	13.5 (10.4–16.6)	12.8 (9.9–16.7)	1.202 (1.199–1.205)
Thailand	2008	1	140	13	9.3 (4.5–14.1)	9 (5.1–16)	1.121 (1.119–1.124)
Thailand	2012	1	47	2	4.3 (-1.5–10)	4.4 (1.1–18.1)	1.002 (1.000–1.005)
Thailand	2013	1	692	82	11.8 (9.4–14.3)	13.7 (10.8–17.2)	0.975 (0.973–0.978)
Thailand	2015	2	1221	108	8.8 (7.3–10.4)	10.6 (8.7–12.9)	0.908 (0.906–0.911)
Togo	2013	1	51	9	17.6 (7.2–28.1)	8.9 (4.3–18.2)	2.359 (2.345–2.374)
Uruguay	1999	1	200	49	24.5 (18.5–30.5)	111.3 (80.6–153.8)	0.291 (0.284–0.297)
US	1997	1	392	137	34.9 (30.2–39.7)	190.7 (154.9–234.7)	0.281 (0.280–0.282)
US	1998	2	482	91	18.9 (15.4–22.4)	81.0 (64.5–101.7)	0.287 (0.286–0.287)
US	2000	1	538	161	29.9 (26.1–33.8)	138.9 (115.5–167.1)	0.306 (0.306–0.307)
US	2004	2	141	30	21.3 (14.5–28)	82.3 (55.0–123.2)	0.327 (0.327–0.328)
US	2005	3	852	81	9.5 (7.5–11.5)	31.1 (24.8–39.2)	0.336 (0.335–0.337)
US	2007	1	223	25	11.2 (7.1–15.4)	37.4 (24.7–56.7)	0.337 (0.336–0.337)
US	2009	2	602	195	32.4 (28.7–36.1)	134.8 (113.7–159.9)	0.354 (0.353–0.355)
US	2010	3	16943	664	3.9 (3.6–4.2)	10.8 (10–11.7)	0.375 (0.374–0.376)
US	2011	2	657	75	11.4 (9–13.8)	33.5 (26.3–42.6)	0.383 (0.383–0.384)
US	2012	1	292	13	4.5 (2.1–6.8)	11.8 (6.8–20.7)	0.392 (0.391–0.392)
US	2013	3	729	197	27 (23.8–30.2)	94.1 (79.9–110.8)	0.392 (0.391–0.393)
US	2016	2	380	148	38.9 (34–43.9)	166.6 (135.5–204.7)	0.382 (0.381–0.382)
US	2003	1	192	16	8.3 (4.4–12.2)	18.9 (11.3–31.5)	0.479 (0.478–0.480)
Vietnam	2004	1	75	5	6.7 (1–12.3)	24.7 (10–61.1)	0.289 (0.287–0.290)
Vietnam	2015	1	205	37	18 (12.8–23.3)	68.4 (47.9–97.6)	0.321 (0.320–0.322)
Overall	-	-	-	-	19.9 (14.7–25.1)*	66.0 (51.4–84.8)	-

Note. Heterogeneity: *Q* = 6327.25, *df* = 86, *p* < .0001, *I*^2^ = 98.63%.

*Overall prevalence was calculated by direct standardization based on country-year weights used in meta-analysis.

**Table 3:** Meta-analysis of HIV prevalence in trans masculine individuals compared to all adults (age 15+).

Country	Year of data collection	Number of samples	Sample size	Frequency of HIV among TM in the samples	Prevalence (95% CI)*	Odds Ratio (95% CI)	HIV prevalence in adults (95% CI)
Australia	2004	1	17	0	0.0 (0.0–0.2)	28.3 (1.7–470.5)	0.101 (0.099–0.102)
Australia	2012	1	28	1	3.6 (-3.3–10.4)	29.4 (4–216.5)	0.126 (0.124–0.127)
Brazil	2002	1	16	0	0.0 (0.0–0.2)	9.7 (0.6–160.9)	0.313 (0.312–0.314)
Brazil	2006	1	51	0	0.0 (0.0–0.1)	2.6 (0.2–42.1)	0.372 (0.371–0.373)
Canada	2013	1	11	0	0.0 (0.0–0.3)	19.1 (1.1–323.8)	0.227 (0.226–0.229)
Italy	2007	1	20	0	0.0 (0.0–0.2)	11.6 (0.7–191.9)	0.210 (0.208–0.211)
Italy	2009	1	27	0	0.0 (0.0–0.1)	8.6 (0.5–140.9)	0.211 (0.210–0.212)
Spain	2008	1	92	2	2.2 (-0.8–5.2)	6.7 (1.6–27.1)	0.332 (0.330–0.334)
US	1997	1	123	2	1.6 (-0.6–3.9)	5.9 (1.5–23.7)	0.281 (0.280–0.282)
US	2003	1	50	0	0.0 (0.0–0.1)	3.0 (0.2–48.3)	0.331 (0.330–0.332)
US	2005	2	124	2	1.6 (-0.6–3.8)	4.7 (1.2–19.2)	0.345 (0.344–0.345)
US	2007	2	92	8	8.7 (2.9–14.5)	26.2 (12.7–54.2)	0.362 (0.361–0.362)
US	2009	2	292	2	0.7 (-0.3–1.6)	1.8 (0.5–7.3)	0.379 (0.378–0.379)
US	2010	2	5256	21	0.4 (0.2–0.6)	1.0 (0.7–1.6)	0.387 (0.386–0.388)
US	2011	1	120	10	8.3 (3.4–13.3)	22.9 (12–43.8)	0.395 (0.395–0.396)
US	2012	1	30	1	3.3 (-3.1–9.8)	8.7 (1.2–63.7)	0.396 (0.395–0.396)
US	2013	1	122	0	0.0 (0.0–2.8)	1.0 (0.1–16.7)	0.392 (0.391–0.393)
Overall	-	-	-	-	2.56 (0.0–5.9)*	6.8 (3.6–13.1)	-

Note. Heterogeneity: *Q* = 13.06, *df* = 16, *p*<0.0001, *I*^2^ = 70.62%.

*Overall prevalence was calculated by direct standardization based on country-year weights used in meta-analysis.

Standardized prevalence rates and overall odd ratios (based on weights from each country by year) according to geographic region are presented in Table 4. In sub-Saharan Africa (*n* = 1192), standardized HIV prevalence among trans feminine individuals was 29.9% (95% CI 22.5% - 37.3%) and the overall OR for HIV infection, compared to individuals over 15 years of age in the countries from which we had prevalence data for trans feminine individuals, matched to year of data collection, was 21.5 (95% CI 6.3–73.7). In Latin America (*n* = 7917), standardized prevalence was 25.9% (95% CI 20.0% - 31.8%) and the overall OR for HIV infection, compared to individuals over 15 years of age, was 95.6 (95% CI 73.7–122.7). In Asia (*n* = 14,798), the standardized HIV prevalence was 13.5% (95% CI 2.3% - 17.7%) and the overall OR was 68.0 (95% CI 42.9–107.8). Lastly, in the Global North, thus in Australia, Europe, and North America (*n* = 24,697), the standardized HIV prevalence was 17.1% (95% CI 13.1% - 21.1%) and the overall OR for HIV infection was 48.4 (95% CI 28.2–83.9).

**Table 4:** HIV prevalence and odds ratios for trans feminine individuals compared to all adults (age 15+), separated by geographic region.

Region	Number of countries	Number of Samples	Sample size	Prevalence (95% CI) *	Odds Ratio (95% CI) *	HIV prevalence in adults (95% CI)*
Africa	9	9	1192	29.9 (22.5–37.3)	21.5 (6.3–73.7)	4.69 (4.67–4.71)
Asia	11	35	14798	13.5 (2.3–17.7)	68.0 (42.9–107.8)	0.344 (0.343–0.345)
Global North	5	35	24697	17.1 (13.1–21.1)	48.4 (28.2–83.9)	0.297 (0.296–0.298)
Latin America	9	23	7917	25.9 (20.0–31.8)	95.6 (73.7–122.7)	0.391 (0.388–0.394)

Note. The HIV prevalence in adults of the population (last column) is the weighted prevalence of the countries included in this meta-analysis, not overall prevalence in the region.

* Results were calculated by direct standardisation of country-year sample size instead of pooling.

The standardized HIV prevalence by sampling method is reported in Table 5. The standardized HIV prevalence among transgender individuals when respondent driven sampling was employed (33 studies) was 23.3% (95% CI 18.0% - 28.4%). When prevalence rates were ascertained via STI clinic visits (26 studies), standardized prevalence was 17.4% (95% CI 12.2% - 22.7%). When convenience sampling was employed (14 studies), standardized prevalence was 19.7% (95% CI 14.8% - 24.5%) and when cluster sampling was employed (11 studies), the standardized prevalence was 19.6% (95% CI 14.4% - 24.9%). The remaining sampling methods were relatively infrequently employed (i.e., employed in 5 or fewer studies).

**Table 5:** HIV prevalence in trans feminine individuals, separated by sampling method.

Sampling method	Number of samples	Sample size	HIV prevalence (95% CI)*
Respondent driven sampling	33	12202	23.3 (18.0–28.4)
STI clinic visit	26	19360	17.4 (12.2–22.7)
Convenience sampling	14	3733	19.7 (14.8–24.5)
Cluster sampling	11	4273	19.6 (14.4–24.9)
Hospital	5	827	15.0 (9.8–20.4)
Snowball	4	1203	11.8 (8.0–15.6)
Surveillance	4	2339	9.1 (6.1–12.0)
NGO	2	213	37.8 (31.5–44.2)
Database health plan	1	3475	5.4 (4.6–6.1)
Purposive sampling	1	538	29.9 (26.1–33.8)

Note. For one study, the sampling method was unknown and is not included in this table.

*: Results were calculated by direct standardisation of country-year sample size instead of pooling.

**Fig 4:**
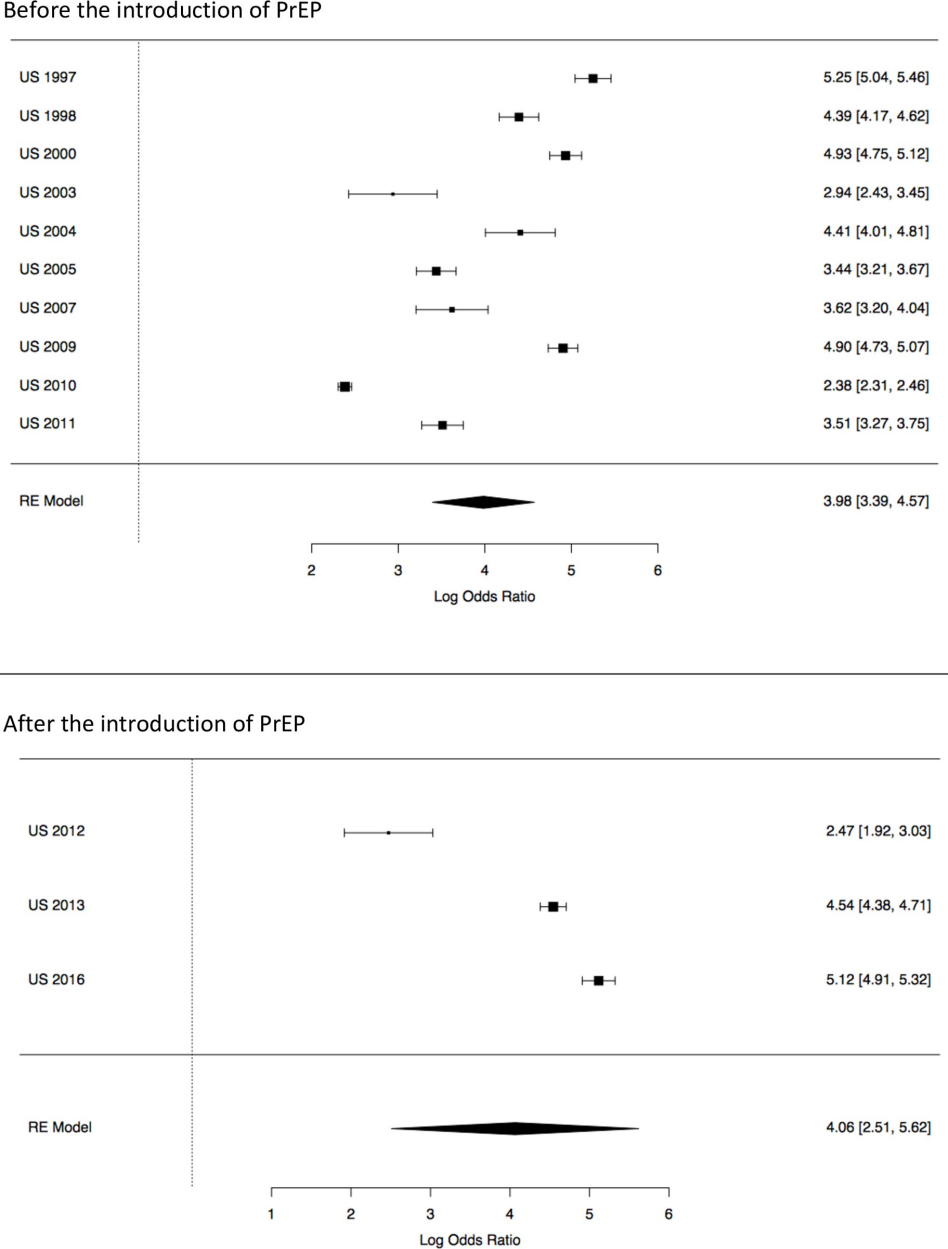
Forest plot of HIV prevalence in trans feminine individuals in the USA compared to all adults (age 15+) in the USA. The 10 country-year including 18 studies above the line are studies where data were collected prior to the introduction of PrEP (2012). The 3 country-year including 6 studies below the line are studies where data were collected after the introduction of PrEP. The scale on the x-axis is log odds ratio. The percentages indicate the weight of each sample within the meta-analysis. The numbers in the right column are the log odds ratios including their confidence intervals.

Next, we looked at the potential role of PrEP in reducing HIV prevalence among trans feminine individuals. Prior to the introduction of PrEP (1997–2011), the standardized HIV prevalence in US-based studies was 18.4% (95% CI 14.8% - 22.0%; Table 6) and the overall OR for HIV infection, compared to individuals over 15 years of age in the USA, was 53.5 (95% CI 29.7–96.5). After the introduction of PrEP (2012–2017), the standardized HIV prevalence in US-based studies was 23.7% (95% CI 20.2% - 27.2%) and the OR for HIV infection, compared to individuals over 15 years of age, was 58.0 (95% CI 12.3–275.9). The forest plot of this analysis is presented in Fig 4.

**Table 6:** HIV prevalence and odds ratios for trans feminine individuals compared to all adults (age 15+) in US-based studies, according to whether data was collected before or after the introduction of PrEP (2012).

	Number of studies	Sample size	Frequency of HIV among TF in the samples	Prevalence (95%CI)*	Odds Ratio (95%CI)
Before PrEP	18	21022	1475	18.4 (14.8–22.0)	53.5 (29.7–96.5)
After PrEP	6	1401	358	23.7 (20.2–27.2)	58.0 (12.3–275.9)

*Note*. *Overall prevalence was calculated by direct standardization based on country-year weights used in meta-analysis.

Heterogeneity was high across the studies that included trans feminine individuals (*Q* = 6327.25, *df* = 86, *p* < .0001, *I*^2^ = 98.63%). This may be because the studies were conducted in different countries using different methodologies. Heterogeneity was moderate for the studies that included trans masculine individuals (*Q* = 102.06, *df* = 15, *p*<0.0001, *I*^2^ = 72.17%). The funnel plots showed an asymmetrical distribution of studies and may therefore indicate publication bias (S2 Appendix in Fig A2.1 and Fig A2.2).

## Discussion

This systematic review and meta-analysis affirms that transgender individuals are disproportionately burdened by HIV, and that this is the case for not only trans feminine individuals, but also for trans masculine individuals. Using a larger pooled sample than ever compiled before, we ascertained that trans masculine individuals almost seven times more likely to have HIV, and trans feminine individuals are 66 times more likely to have HIV, than other individuals over 15 years of age. Additionally, based on data from 34 countries across major geographic regions, we found support for the contention that the disproportionate burden for HIV carried by transgender individuals is a worldwide phenomenon, and that some regions, such as Africa and Latin America, may be impacted more than others. Further, we established that sampling methods are likely to impact prevalence rates and that, to date, PrEP prevention effects on HIV prevalence cannot be established.

To our knowledge, no previous study has estimated the HIV burden carried by trans masculine individuals worldwide. Reisner and Murchinson [1] did conduct a research synthesis of HIV risks in trans masculine individuals where laboratory-confirmed prevalence ranged from 0% to 4.3% and Becasen and colleagues [17] established a laboratory-confirmed estimated prevalence rate of 3.2% but, in both studies, no odds ratios were calculated to ascertain the relative burden of HIV carried by trans masculine individuals. Our finding that trans masculine individuals are almost seven times more likely to have HIV than other individuals over 15 years of age indicates that many trans masculine individuals are indeed at risk for HIV. The presumption that trans masculine individuals almost exclusively have sex with cis-gender women and are therefore not at risk for HIV is thus incorrect [1]. As indicated by Reisner and Murchinson, there is a diverse range of bio-anatomies represented among trans masculine individuals and their partners in sexual encounters, and these should be considered in HIV prevention efforts [1].

Our finding that trans feminine individuals are 66 times more likely to have HIV than other individuals over 15 years of age is a higher estimate that the estimate generated in Baral and colleagues’ meta-analysis, [4] where the odds ratio for HIV infection among transgender women was 49.1. We believe that the odds ratio and prevalence rates established in our systematic review and meta-analysis are likely more realistic estimations for two reasons. First, our methodological approach used standardized rather than pooled prevalence and took into account not only country but also year of data collection. In a pooled estimate, the total study population and total HIV cases are summed, and then a crude proportion is calculated. This does not take heterogeneity and variation among the included studies into account. Our standardization approach entailed taking the weights from each country-year into account. Without the weighted standardization, a country-year combination that contains large or small study samples is likely to deliver misleading pooled results. The standardized approach thus delivers a more robust estimation than a pooled approach. Second, due to recent increases in the number of studies reporting HIV prevalence among transgender individuals, the total sample of transgender individuals in our meta-analysis was almost four and a half times larger than the pooled sample in Baral et al. [4] Third, the data reviewed in Baral et al. was derived from 15 countries, all of which have male-dominant epidemics, while the data in the meta-analysis reported here was derived from 34 countries, thus lending additional support to the contention that the high burden of HIV among transgender individuals is a worldwide phenomenon.

Our finding that HIV prevalence among transgender individuals appears to be, over the course of the epidemic, higher in African and Latin American regions may point to greater disapproval of gender fluidity and the accompanying marginalization that puts transgender individuals more at risk for HIV in these regions, although we recognize that overall prevalence rates for HIV are higher in Sub-Saharan Africa than in many other regions. Nonetheless, this was the first systematic review and meta-analysis to include samples from Sub-Saharan Africa, and the findings from Sub-Saharan Africa point to a significant burden of HIV among transgender individuals. However, given that, in our analyses, the sample sizes for African regions and Latin America were smaller than the sample sizes for other regions, more research is needed to confirm that transgender individuals in these regions do indeed have higher prevalence rates and carry an even greater burden of HIV. Additionally, future research should also seek to establish HIV prevalence rates and burdens in other understudied regions such as Eastern Europe.

This meta-analysis also demonstrated that sampling methods are likely to impact prevalence rates. This is in line with critiques of sampling methods that were levied in earlier commentaries on Baral et al. [4] and in other studies [11, 18, 134]. In our study, the various sampling methods generated very different prevalence rates for HIV in trans feminine individuals ranging from 5.4% to 37.8%. However, the four most frequently used sampling methods, namely respondent-driven sampling, sampling via STI clinics, convenience sampling, and cluster sampling had similar ranges (17.4% to 23.3%). We believe that the impact of sampling methods on prevalence rates is in need of further investigation. In our analyses, unambiguous classification was not always possible and the prevalence rates generated for less common sampling methods may be less reliable. We therefore recommend more comprehensive investigations of the impact of sampling methods in transgender studies.

In our meta-analyses, we also explored the potential role of PrEP availability by comparing studies conducted in the US where data was collected prior to and after the introduction of PrEP. No effect of PrEP could be established yet in our analyses. In fact, we found a higher HIV prevalence rate following the introduction of PrEP. This may be because there were only six studies done following the introduction of PrEP and the total sample after PrEP introduction was smaller and possibly less representative than the 18 studies conducted before PrEP was introduced. It is possible that no reduction in prevalence due to PrEP is the result of PrEP not yet reaching trans individuals. The inclusion of transgender individuals in PrEP trials has been low and access to PrEP for transgender individuals has been limited.[20, 21, 135] However, a qualitative study on PrEP acceptability among transgender women in San Francisco showed that interest was relatively high once participants were informed about the possibilities, thus suggesting that transgender individuals at high risk for HIV need to be informed about PrEP [20]. By the same bio-medical token, future meta-analytic studies should also include Treatment-as-prevention (TasP) effects in their analysis, once sufficiently robust primary data is available.

This systematic review and meta-analysis should be interpreted in light of possible limitations. One is potential sample size biases for studies originating from countries other than the USA, and those of trans masculine individuals. To be able to present a comprehensive, global picture, we set a lower bound for trans feminine individuals, excluding sample sizes of trans feminine individuals less than 40. Yet, we did not apply a minimum sample size for studies among trans masculine individuals as this would have resulted in the exclusion of most studies reporting HIV prevalence among trans masculine individuals. Further, we were not in a position to conduct city-level comparisons and thus acknowledge that our country-level analyses may provide a less precise estimation of the odds ratios, as these do not take into account that, in some countries, the HIV epidemic is more concentrated in certain areas. A third possible limitation is related to our classification of sampling methods. Unambiguous classification was not always feasible and it is possible that the prevalence rates generated for less common sampling methods were less reliable. Fourth, in our meta-analysis, the sample sizes for African regions and Latin America were smaller than the sample sizes for other regions, and this may have impacted the prevalence rates. Additionally, no prevalence rates from Eastern Europe were available. Fifth, our analysis did not account for sexual orientation or the presence or absence of gender reassignment surgery, both of which can impact HIV risk. It is also did not separately ascertain prevalence rates for trans feminine individuals who engage in sex work versus those who do not as primary level data on this is not available on a global scale. We recommend that future research take these potential shortcomings into consideration. Specifically, we recommend that future research explicitly investigate prevalence among sub-populations within the transgender community, and that new studies also take changes in HIV treatment (TasP) and sampling strategies, as well as their interactions, into account, as this will provide an even more comprehensive picture of HIV prevalence and burden among transgender individuals.

### Summary and recommendations

That transgender individuals, both trans feminine and trans masculine, are, worldwide, disproportionately burdened by HIV points to the need to pay explicit attention to the unique HIV prevention and care needs of transgender individuals. HIV surveillance and research has traditionally grouped transgender individuals, particularly trans feminine individuals, with men who have sex with men (MSM), thus conflating gender with anatomy. This obscures the unique situation and vulnerabilities to HIV of transgender people [100]. It is therefore necessary to abandon the aggregation of data across MSM and trans feminine women [100, 136]. Additionally, and in line with MacCarthy et al., [11] we also propose disaggregating data across trans feminine and trans masculine individuals [106]. Although some individual and structural risk factors for HIV may be shared by transgender individuals, trans feminine and trans masculine individuals have unique needs [106].

Ascertaining that transgender individuals continue to be disproportionately burdened by HIV is important as it can serve as an impetus for efforts to change this burden. Although transgender individuals in certain regions may more affected by HIV and knowing that transgender processes are diverse across the world, we contend that it is important to, in all regions, target multiple levels of HIV risk, as well as their antecedents and their intersections, while being cognizant of the local context. Targeting individual level risk factors, such as unprotected sex, STI co-infection, and needle sharing, must occur alongside broader efforts to support transgender individuals and reduce stigmatization and marginalization [9, 137, 138].

Paramount to HIV risk reduction is gender affirmation, and in the context of HIV, gender affirmation is particularly important in health care [139–141]. Discrimination, judgment, insensitivity, and a lack of understanding from health care providers prevents many transgender individuals from accessing HIV prevention, testing, treatment, and care services [142]. Gender affirming care is not simply the provision of hormones and gender-affirming surgeries; it also includes using patients’ preferred names and pronouns, respecting diversity in gender identities and expressions, employing inclusive intake forms, displaying images that are welcoming to transgender individuals, and creating safe spaces where transgender individuals can be themselves [135, 143]. We recommend integrating HIV prevention and care services in broader gender-affirming care services [6, 135, 142]. This includes actively making PrEP available to transgender individuals [141, 144, 145].

## Conclusion

In sum, this systematic review and meta-analyses have served to update our understanding of HIV prevalence over the course of the epidemic as well as HIV burden in both trans feminine and trans masculine individuals using a larger sample than ever before, and has shown that, worldwide, both carry a substantially higher burden of HIV than other individuals over 15 years of age. It has further demonstrated that a by country and year analysis is recommended, that prevalence rates are higher in African and Latin American regions, that sampling methods may impact prevalence rates, and that, at this point in time, the evidence does not suggest that PrEP has played a role in reducing HIV among transgender individuals.

## Supporting information

S1 Appendix Initial meta-analyses. (DOCX)

S2 Appendix Fig A2.1. Funnel plot for the countries describing data from trans feminine individuals. Fig A2.2. Funnel plot for the countries describing data from trans masculine individuals.
(ZIP)

S1 Checklist PRISMA 2009 checklist. (DOC)
