# Supplementary material for: The worldwide burden of HIV in transgender individuals: An updated systematic review and meta-analysis
Source: PLoS One. 2021 Dec 1;16(12):e0260063. doi: 10.1371/journal.pone.0260063 (PMC8635361; doi:10.1371/journal.pone.0260063)
Supplement: S1 Appendix — (DOCX) [file pone.0260063.s001.docx]

**Appendix 1**

This appendix presents the results of the initial meta-analyses conducted using analogous methodology to prior meta-analyses (4,26,27). As indicated, for this analysis, we grouped studies by country, weighted by sample size. We then calculated pooled HIV prevalence and 95% confidence intervals (CIs) per country separately for trans feminine and trans masculine samples. In line with previous meta-analyses, we then calculated odds ratios per country by dividing the HIV prevalence among transgender individuals (numerator) by the HIV prevalence rate among individuals over 15 years of age in the general population in the country from which the sample was derived (denominator), as reported by the 2017 UNAIDS reports (where prevalence estimates for adults are from 15 years of age onward) (28) and estimations of adult population size from the US Census Bureau International Division (29). Thus, in contrast to the main results described in Tables 2 and 3 in the article, the analyses in this appendix are not corrected for year of data collection.

In this analysis, the overall pooled HIV prevalence was 13.0% (95% CI 12.7% - 13.3%; Table A1.1) for trans feminine individuals and 0.8% (95% CI 0.6% - 1.0%; Table A11.2) for trans masculine individuals. The pooled OR for HIV infection, compared with individuals over 15 years of age, was 38.1 (95% CI 26.9 - 53.7; Table A1.1) for trans feminine individuals and 3.5 (95% CI 1.2 - 10.0; Table A1.2) for trans masculine individuals.

**Table A1.1: Meta-analysis of HIV prevalence in trans feminine individuals compared to all adults (age 15+).**

| **Country** | **Number of samples** | **Sample size** | **Prevalence (%)** | **Prevalence (%) CI** | **Odds Ratio** | **Odds Ratio CI** | **HIV prevalence in adults (%)** |
| --- | --- | --- | --- | --- | --- | --- | --- |
| Argentina | 3 | 490 | 33.5% | 30.5% - 36.5% | 134.3 | 121.5 - 160.8 | 0.36% |
| Australia | 2 | 218 | 6.4% | 3.2% - 9.7% | 50.4 | 29.4 - 86.5 | 0.14% |
| Bangladesh | 1 | 889 | 0.8% | 0.2% - 1.4% | 69.4 | 33.1 - 146.9 | 0.01% |
| Brazil | 7 | 4267 | 30.5% | 29.1% - 31.9% | 83.1 | 77.5 - 88.2 | 0.53% |
| Burkina Faso | 1 | 108 | 2.8% | -0.3% - 5.9% | 3.6 | 1.1 - 11.1 | 0.80% |
| Cambodia | 2 | 2266 | 5.2% | 4.3% - 6.1% | 9.6 | 7.9 - 11.5 | 0.57% |
| China | 1 | 498 | 7.6% | 5.3% - 10.0% | 188.7 | 135.6 - 262.4 | 0.04% |
| Colombia | 1 | 58 | 13.8% | 4.9% - 22.7% | 38.5 | 18.2 - 80.6 | 0.41% |
| Côte d’Ivoire | 1 | 298 | 25.5% | 20.6% - 30.5% | 10.6 | 8.2 - 13.7 | 3.12% |
| El Salvador | 1 | 67 | 19.4% | 9.9% - 28.9% | 45.6 | 24.8 - 83.9 | 0.52% |
| India | 7 | 1497 | 18.2% | 16.2% - 20.1% | 98.5 | 86.5 - 112.2 | 0.23% |
| Indonesia | 5 | 1783 | 27.1% | 25% - 29.2% | 116.7 | 105.6 - 129 | 0.32% |
| Italy | 3 | 702 | 24.6% | 21.5% - 27.8% | 134.3 | 113.3 - 159.2 | 0.24% |
| Jamaica | 1 | 103 | 25.2% | 16.9% - 33.6% | 20.3 | 12.9 - 31.5 | 1.64% |
| Lebanon | 1 | 40 | 10.0% | 0.7% - 19.3% | 249.6 | 89.1 - 706.3 | 0.04% |
| Lesotho | 1 | 71 | 59.2% | 47.7% - 70.6% | 5.0 | 3.1 – 8.0 | 22.57% |
| Malawi | 1 | 75 | 16.0% | 7.7% - 24.3% | 1.8 | 1.0 - 3.4 | 9.42% |
| Malaysia | 1 | 193 | 12.4% | 7.8% - 17.1% | 37.0 | 24.0 - 56.3 | 0.38% |
| Mali | 1 | 165 | 22.4% | 16.1% - 28.8% | 22.0 | 15.3 - 31.8 | 1.29% |
| Mexico | 1 | 100 | 22.0% | 13.9% - 30.1% | 111.1 | 69.4 - 179.5 | 0.25% |
| Netherlands | 1 | 69 | 18.8% | 9.6% - 28.1% | 144.0 | 79.0 - 262.4 | 0.16% |
| Nigeria | 1 | 105 | 71.4% | 62.8% - 80.1% | 83.9 | 54.6 - 127.7 | 2.90% |
| Pakistan | 7 | 3669 | 5.0% | 4.3% - 5.6% | 52.5 | 45.6 - 59.7 | 0.10% |
| Paraguay | 1 | 237 | 27.0% | 21.4% - 32.7% | 101.5 | 75.9 - 135.6 | 0.36% |
| Peru | 6 | 1954 | 21.7% | 19.9% - 23.5% | 89.1 | 79.8 - 99.5 | 0.31% |
| Philippines | 1 | 299 | 3.7% | 1.5% - 5.8% | 38.9 | 21.3 - 70.8 | 0.10% |
| Senegal | 1 | 199 | 37.2% | 30.5% - 43.9% | 133.0 | 99.5 - 177.7 | 0.44% |
| Spain | 5 | 1285 | 19.2% | 17.1% - 21.4% | 65.4 | 56.8 - 75.2 | 0.36% |
| Swaziland | 1 | 120 | 14.2% | 7.9% - 20.4% | 0.4 | 0.3 - 0.7 | 26.99% |
| Thailand | 6 | 2574 | 10.5% | 9.3% - 11.6% | 15.0 | 13.2 - 16.9 | 0.77% |
| Togo | 1 | 51 | 17.6% | 7.2% - 28.1% | 10.7 | 5.2 - 22.0 | 1.96% |
| Uruguay | 1 | 200 | 24.5% | 18.5% - 30.5% | 66.7 | 48.4 - 91.8 | 0.48% |
| US | 24 | 22423 | 8.2% | 7.8% - 8.5% | 17.6 | 16.9 - 18.5 | 0.50% |
| Vietnam | 2 | 280 | 15.0% | 10.8% - 19.2% | 54.1 | 38.9 - 74.4 | 0.33% |
| **Pooled estimate*** |  | **47353** | **13.0%** | 12.7% - 13.3% | **38.1** | **26.9 - 53.7** | **..** |

*Note.* * Heterogeneity *Q*=4293.39, *df*=33, *p*<.001, *I*^2^=99.2%.

**Table A1.2: Meta-analysis of HIV prevalence in trans masculine individuals compared to all adults (age 15+).**

| **Country** | **Number of samples** | **Sample size** | **Prevalence (%)** | **Prevalence CI (%)** | **Odds Ratio** | **Odds Ratio CI** | **HIV prevalence in adults (%)** |
| --- | --- | --- | --- | --- | --- | --- | --- |
| Australia | 2 | 45 | 2.2% | -2.1% - 6.5% | 16.6 | 2.3 - 121.5 | 0.14% |
| Brazil | 2 | 67 | 0.0% | 0% - 5.4% | 1.4 | 0.1 - 22.7 | 0.53% |
| Italy | 2 | 47 | 0.0% | 0% - 7.6% | 4.3 | 0.3 - 70.1 | 0.24% |
| Spain | 1 | 92 | 2.2% | 0% - 5.2% | 6.1 | 1.5 - 24.8 | 0.36% |
| US | 13 | 6209 | 0.7% | 0.5% - 1% | 1.5 | 1.1 – 2.0 | 0.50% |
| **Pooled estimate*** | **20** | **6460** | **0.8%** | 0.5% - 1% | **3.5** | 1.2 – 10.0 | **..** |

*Note.* * Heterogeneity *Q*=9.57, *df*=4, *p*=.023, *I*^2^=58.2%. Since the HIV prevalence was 0 in Brazil and Italy, we estimated the CI for the prevalence using the Wilson Score Interval.(128)
