# Supplementary figures and images for: The worldwide burden of HIV in transgender individuals: An updated systematic review and meta-analysis
Source: PLoS One. 2021 Dec 1;16(12):e0260063. doi: 10.1371/journal.pone.0260063 (PMC8635361; doi:10.1371/journal.pone.0260063)

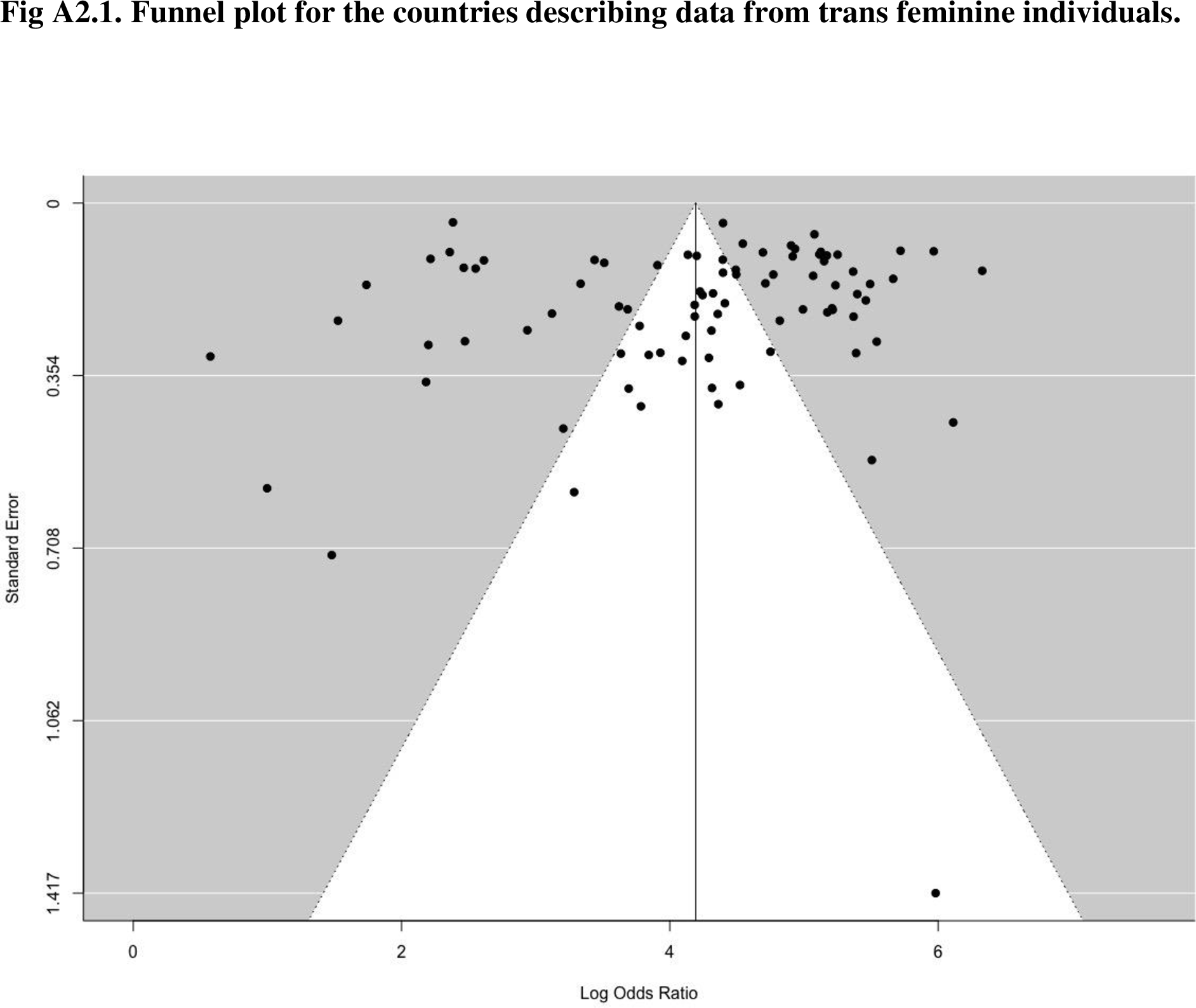

Supplement: S2 Appendix — Fig A2.1. Funnel plot for the countries describing data from trans feminine individuals. Fig A2.2. Funnel plot for the countries describing data from trans masculine individuals. (ZIP) [file pone.0260063.s002.zip › FigA2.1.tif]

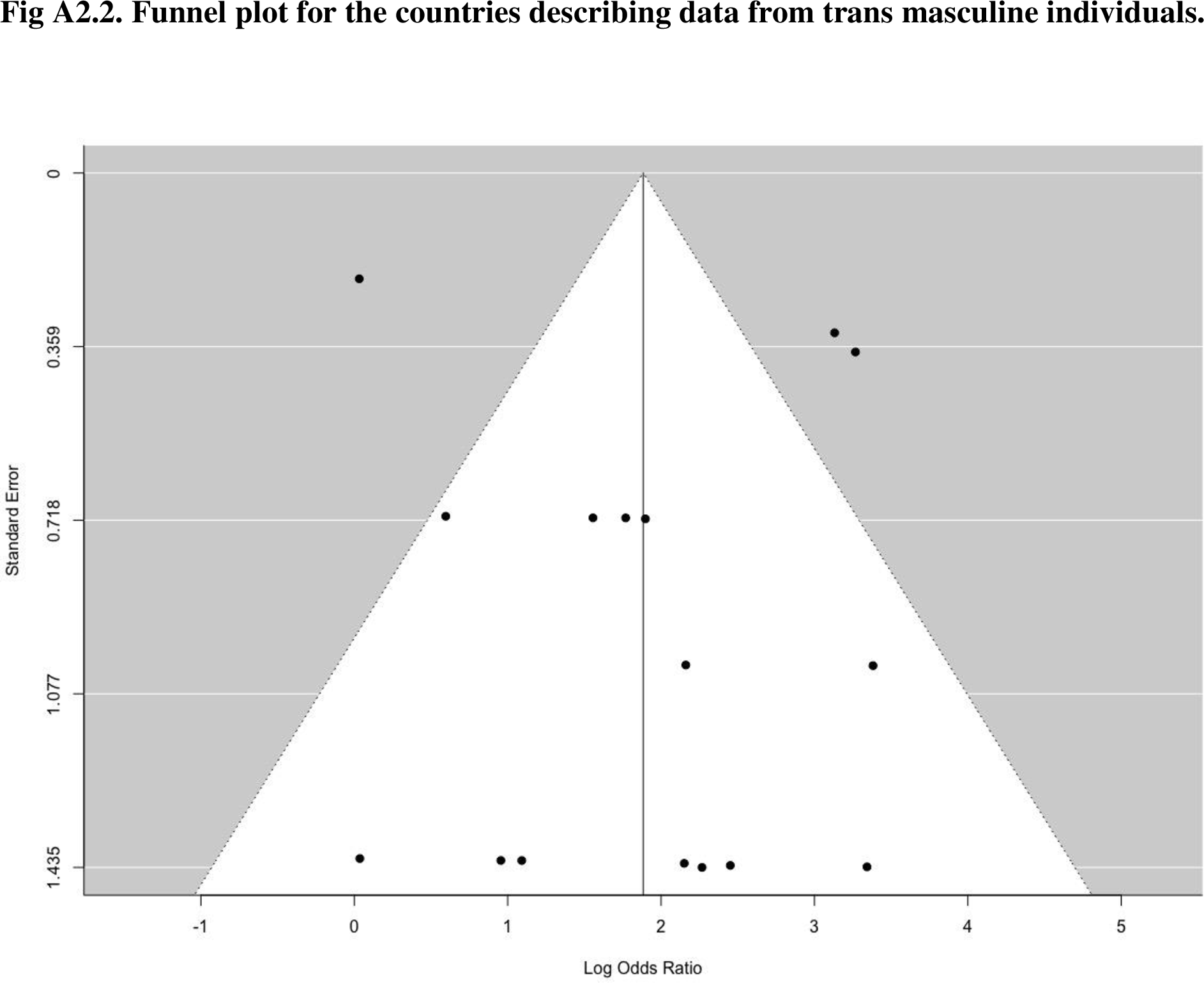

Supplement: S2 Appendix — Fig A2.1. Funnel plot for the countries describing data from trans feminine individuals. Fig A2.2. Funnel plot for the countries describing data from trans masculine individuals. (ZIP) [file pone.0260063.s002.zip › FigA2.2.tif]
