# Supplementary material for: The worldwide burden of HIV in transgender individuals: An updated systematic review and meta-analysis
Source: PLoS One. 2021 Dec 1;16(12):e0260063. doi: 10.1371/journal.pone.0260063 (PMC8635361; doi:10.1371/journal.pone.0260063)
Supplement: Attachment — Submitted filename: 2020-05-05 Response to reviewers.docx [file pone.0260063.s004.docx]

May 5^th^, 2020

Dear Dr. Wei,

Thank you for providing us with the opportunity to revise our manuscript entitled, ‘The worldwide burden of HIV in transgender individuals: An updated systematic review and meta-analysis’ that we previously submitted for consideration in PLOS ONE’s **special issue on health inequities and disparities**. We appreciate the feedback from the reviewers and believe that our revised manuscript is an improvement on the original. You will find a comprehensive response to the reviewers’ comments below.

We hope that our manuscript now matches the standards and interests of PLOS ONE’s special issue on health inequities and disparities, and that it will become a substantial and important contribution to a better understanding of health inequalities, particularly as they pertain to transgender individuals and HIV burden.

We look forward to hearing from you soon.

Kind regards,

On behalf of my co-authors,

Sarah Stutterheim

***Reviewer 1***

*1) Given the number of systematic reviews and meta-analyses on HIV prevalence in this population, including within the last 5 years, additional rationale for embarking on this study could be presented in the Introduction. What questions are the authors asking and answering that previous work has not covered, and why is it important? This is especially relevant given that the search time frame goes back so far, and prior studies have incorporated much of the pre-2015 studies included here.*

Thank you for this comment. The introduction in the revised manuscript outlines why a comprehensive, updated systematic review and meta-analysis is required. We argue that an updated review is needed because HIV risk is dynamic, particularly for transgender individuals (pg. 4). We also outline limitations of previous reviews, particularly those that have been published more recently. For example, Reisner and Murchison (2016) focused only on HIV prevalence in transmasculine individuals. Our systematic review and meta-analysis focuses on both transmasculine and transfeminine individuals. Poteat and colleagues’s (2016) article comprised only a systematic review and not a meta-analysis, and the same applies for MacCarthy et al (2017). Furthermore, MacCarthy et al. provide only very limited insight on HIV prevalence as the inclusion criteria for their systematic review required that studies reported both HIV and STI prevalence, which yielded only six studies. Lastly, Becasen and colleagues (2019) systematic review and meta-analysis focused only on HIV prevalence in the US while our looks at the worldwide burden of HIV infection. Our systematic review and meta-analyses is therefore more comprehensive that other reviews published since Baral et al. (2013). We make this explicit on page 6 of the revised manuscript. Also, we outline that our updated systematic review and meta-analysis further adds to the literature by exploring sampling frames and the potential impact of PrEP (pg. 6).

With regards to the time frame chosen, we followed Baral et al. (2013) in the our approach to this systematic review and meta-analysis as we felt this would enable clearer comparison between what was ascertained then and what we ascertained in our updated review. We considered this to be more informative than choosing a time frame after Baral et al’s publication. In fact, we believe that the longer time frame provides a more comprehensive and nuanced picture of HIV prevalence and burden for transgender individuals. We now state this explicitly under ‘Search strategies and eligibility’ on page 7.

*2) The use of a country-specific, general population HIV prevalence rate to make comparisons and generate odds ratios is an innovative approach, but it requires greater clarification and specificity in the Methods. The vast majority of the studies included are not country-level (or even city-level), but rather much more local. This makes the comparison group somewhat spurious, as the cities wherein trans people's HIV prevalence was assessed will in almost all cases have higher underlying HIV prevalence rates in the general population than will each respective country as a whole. Furthermore, it is unclear whether the country-level HIV prevalence rates are aligned with the sampling year(s) of each respective study: if they are not (and I can't tell here), then they probably need to be readjusted to the respective study year before generating OR.*

Thank you for this comment. We acknowledge that using country-specific, general population HIV prevalence to make comparisons and generate odd ratios may be somewhat limited in terms of precision, particularly in contexts where city-level rates differ from country-level rates. While we considered the possibility of presenting the data at the city-level instead of country-level, we choose not to do this. We felt that that it would be more parsimonious to present the data at a country level. Country level data are, in our opinion, more useful as this data allowed for the same calculation for every country, thus increasing the comparability between countries. The aim of a meta-analysis is to provide a general overview, and presenting data at a city-level would have added substantial additional complexity that would undermine the ultimate aim of the meta-analysis. Furthermore, city-level data were often not available (many countries collect data nationally rather than per city) and there were many studies included that derived prevalence rates across various cities within a country. Additionally, for most countries, we combined multiple studies from several cities, thus further justifying our country-level approach.

This country-level approach has previously been employed and we now explicate this on page 10 of the revised manuscript, referring to three prior meta-analyses that used this approach. We also immediately acknowledge, in the text, the limitations of this approach. To wit: “we acknowledge that this approach may provide a less precise estimation of the odds ratios, as it does not take into account that, in some countries, the HIV epidemic is more concentrated in certain areas.” (pg, 10).

Thanks also for pointing out that we neglected to state the year upon which we based country-level HIV prevalence rates. We now make explicit that this was 2017 on page 10 of the revised manuscript. We did not adjust these rates based on sampling years of the studies for two reasons. Firstly, in most countries, HIV prevalence in the general population has been quite stable over time. As a result, we do not expect that adjustment based on sampling year would have a large effect. Secondly, we believe that this adjustment would have made analyses unnecessarily complex, and potentially biased. Many of the studies included in our meta-analysis sampled over multiple years, while others only covered shorter periods. As a consequence, averaged and non-averaged data would have been the result. In addition, and related to previous paragraph, adjusting also for sampling year would have required the calculation of odds ratio *per country per year*, making the results even more complicated and less parsimonious. In sum, we believe that while adding this level detail to the analysis may contribute to the overall quality of results, it would unfortunately render them fragmented.

*3) Please consider using study year as a moderator of HIV prevalence throughout. I appreciate the example shown with PrEP in U.S.-based studies; but the rollout of ART, PEPFAR, and subsequent treatment-as-prevention policies likely have a greater effect on HIV prevalence than PrEP does, including among trans people. It would be helpful to sampling year(s) as a moderating variable throughout these analyses, for instance using 5-year periods (2000-2004; 2005-2009, etc) to categorize moderating effects. Without this, you cannot show trends in HIV infection in this population as clearly; and more importantly, it is hard to tell where we are now (compared to where we were 20 years ago).*

We thank the reviewer for this suggestion. At the outset of this meta-analysis, we considered including study year as a moderator but decided not to do so for several reasons. Firstly, not every manuscript provides study year information and obtaining additional information on published studies from authors has proven to be difficult. Secondly, had we had been able to acquire that information (and our experience was that we could not), we would have ended up with many studies that do not fit in 5-year periods unanimously for all countries. The periods would then differ by country and also reduce, due to exclusion, sample sizes even more. In that sense, such an endeavor would make Reviewer 2’s comment (see below) even more valid. Thus, we aimed at maintaining the most robust data (in terms of sample size) with a global perspective. We agree that this limits the potential of the data to show trends in HIV infection as clearly as we would like to, but we had to work with the information that is available. If we had taken the approach suggested by the reviewer, we would have only been able to report data from the US, which is clearly not desirable from a global perspective. Nonetheless, we have now included not using study period as a moderator as a limitation on page 28 of the manuscript.

*4) Typo "indivi" on p. 25, line 121.*

Thanks for showing us this typo. It has been corrected in the revised manuscript.

**Reviewer 2**

*I reviewed the statistical approach used in this paper only. The approach that was used is suitable, with one caveat: In Table 3, sample sizes from countries outside the US appear too small to estimate prevalence. Can the authors comment on the acceptability of using such small samples?*

We thank the reviewer for this comment. In the revised manuscript, we have added/expanded on small sample sizes as a limitation (see pg. 28). We do believe that including the data from countries outside of the US is necessary if we aim to provide a comprehensive and global picture of HIV prevalence and burden. Otherwise, the meta-analysis would be US-centric, and this would lead to different type of criticism. In this double trade-off situation, we believe that it is more important to report global data, and this is in line with previous approaches (see Baral et al., 2013). We hope the reviewer concurs.
