# Supplementary material for: The worldwide burden of HIV in transgender individuals: An updated systematic review and meta-analysis
Source: PLoS One. 2021 Dec 1;16(12):e0260063. doi: 10.1371/journal.pone.0260063 (PMC8635361; doi:10.1371/journal.pone.0260063)
Supplement: Attachment — Submitted filename: 2020-11-30 Response to reviewer.docx [file pone.0260063.s005.docx]

*Please address major weaknesses identified by reviewer #1, in particular his comments # 1 & 2.*

*1) Please reconsider extracting sampling year(s) from each study, as best as is possible given that it may not be provided in some cases (please show from what studies this is not provided). That data can be incorporated into Table 1*.

Thank you for this comment. Sampling years are included in Table 1. In previous versions, this information was included in Table 1 as well, in the final column with the title ‘Year of data collection’. In this revised version, we have moved this column to the left so that it is more evident. It is now placed next to the ‘Year of publication’ column.

*2) If not accepting a city-level comparison, please at least consider using study-specific, country-level data for the general population comparison group for the year (or, mean rate for multiple years if the sampling spans multiple years) that are best aligned with the study's sampling year(s), given the available UNAIDS and other country-level data.*

Thank you for this comment. As per the reviewer’s request, we have, in our revised manuscript, aligned the years of data collection for each study with country-level prevalence rates for the year(s) in which the data were collected. We have updated the methods section (lines 201-206) accordingly, outlining exactly how this was done. We indicate that we grouped studies by country and year of data collection. To calculate the HIV prevalence in the general population, we used the UNAIDS reports (for HIV frequency estimates) and US Census data (for population estimates) of the corresponding years. In addition, we indicate that we standardized prevalence rates instead of pooled prevalence rates, as this takes the weights for each study into account rather than crudely pooling prevalence rates across studies. Overall, this approach has less bias.

We appreciate the reviewer re-emphasizing these analyses as the more fine-grained analyses do better justice to the data, and we now also mention this in our introduction (lines 110-112). The new analyses have yielded a higher odds ratio for both trans feminine individuals (66.0 versus 38.1 in our previous analysis) AND trans masculine individuals (6.6 versus 3.5). We have therefore adjusted the discussion section to reflect these higher odds ratios and their implications.

Also, as our initial intent was to, using analogous methodology, offer an updated meta-analysis that could be compared with previous meta-analyses, specifically Baral et al. (2013), we have, in our revision, opted not to discard the previous analysis but rather move it to the appendix so that readers can still compare those findings to previous meta-analytical findings. We trust the editor and reviewer agree with this transparency. We also believe that presenting both analyses contributes to both backward comparability and developing a novel standard of meta analytical reporting in this domain.

*3) Please reconsider moderation by study year/period. At the very least, consider using a dichotomous variable classifying moderation by pre- and post- widespread treatment-as-prevention adoption (e.g. ~2012 but will vary by country).*

Thank you for this comment. We agree that such a test of moderation would be desirable, but we chose not to include it in the paper for several reasons. First of all, this analysis is much more complex than the data we are summarizing here: We cannot use country cascade data for the TasP moderation, but we would need cascade data for transgender individuals; such data is not widely available. Secondly, such a sufficiently large amount of data suitable for a meta-analysis would first be available only from the USA, and we value a global focus over a country specific: for some countries we do have only a limited amount of data and a dichotomization would lead to comparing single studies of different size with each other. Third, we ran a moderation analysis for PrEP (which is part of the bio-medical HIV prevention and treatment palette) and it did not yield strong positive findings. In fact, the trend is not yet reversed, but the amount of studies is also rather low. Robustly interpretable data for TasP and PrEP may not be available yet. Finally, the editor had stressed to focus on (1) and (2) of the comments, and we were not convinced, based on our findings, that this analysis could contribute much more to the larger picture. We have added a recommendation in the manuscript that reflects this comment and suggests its incorporation in future studies (lines 409-411). We trust this is acceptable.

*I think this manuscript has substantial potential for impact, which will really be heightened if the comparisons made, and the conclusions and implications that result from these, are less spurious.*
