# Supplementary material for: The worldwide burden of HIV in transgender individuals: An updated systematic review and meta-analysis
Source: PLoS One. 2021 Dec 1;16(12):e0260063. doi: 10.1371/journal.pone.0260063 (PMC8635361; doi:10.1371/journal.pone.0260063)
Supplement: Attachment — Submitted filename: 2021-09-22 Response to reviewers.docx [file pone.0260063.s006.docx]

**REVIEWER 1**

*I commend the authors on their responsiveness to the reviewer comments, which appear to have substantially strengthened this manuscript. Some minor notes for consideration:*

Thank you for your encouragement.

*1) Funding section should be removed from Methods and placed below Acknowledgments.*

Thank you. We have now moved the funding section to below the acknowledgements (lines 492-495).

*2) Because the studies analyzed span so many years, they do not represent current HIV prevalence in trans populations. So I recommend the authors note that as such, and use appropriate language in the abstract and the discussion (e.g., "over the course of the HIV epidemic, mean HIV prevalence among trans women has been X%" rather than "HIV prevalence in trans women is X%".*

Thank you for drawing our attention to the need to hedge and frame claims about prevalence rates. As requested, we have revised the abstract and discussion to reflect this more nuanced claim. We have also reviewed the remainder of the manuscript to ensure that this is abundantly clear throughout. We trust this is now in order.

*3) Some acknowledgment in limitations that moderation by year (including multi-year spans before and after countrywide TasP policies have been promulgated within specific countries) has not been performed, but is likely important for future research: HIV prevalence trends over time is an especially valuable analysis that will tell us a lot about which populations are falling through the structural/policy cracks.*

Thank you for this comment. As indicated in previous rounds of revisions, we agree that a test of moderation would be desirable, but we chose not to include it in the paper for several reasons. First, this analysis is much more complex than the data we are summarizing here: It is not possible to use country cascade data for a TasP moderation; rather, we would need cascade data for transgender individuals, and that data is not widely available. Second, such a sufficiently large amount of data suitable for a meta-analysis would, at this point in time, only be available for the US, and we value a global focus over a country specific focus. For many countries, we have only a limited amount of data and a dichotomization would lead to comparing single studies of different sizes with one another other. Third, we ran a moderation analysis for PrEP (which is part of the bio-medical HIV prevention and treatment palette) and it did not yield strong positive findings. In fact, the trend is not yet reversed, but the amount of studies is also rather low. We thus believe that robustly interpretable data for TasP and PrEP is not yet available. We have added a recommendation in the manuscript that reflects this (lines 437-442) and we trust that this adequately addresses this comment.

*I look forward to seeing this manuscript in print. Thank you for the opportunity to review.*

Thank you! We too are very much looking forward to having this paper in print.

**REVIEWER 3**

*This manuscript is a systematic review and meta-analysis of laboratory confirmed HIV prevalence for transgender populations globally, aimed to update the previously published review by Baral et. al, replicating the methodology. The study is well-conducted and represents an important contribution to the literature. However, there are a number of outstanding major and minor issues with the manuscript currently, as outlined below.*

Thank you for your positive comments and feedback. We agree that this is an important contribution to the literature and hope to have adequately addressed your concerns in this revision.

*• “Sampling frame” is used incorrectly in the paper- the authors seem to be using this to refer to sampling method, however this term refers to the sampling framework or list of individuals in the population from which people were sampled into a given study.*

Thank you for this comment. We have removed the term sampling frame and replaced it with sampling method throughout the paper.

*• What is the rationale for including studies in this review published between 2000-2011- which overlaps directly with previously reviewed literature in the Baral et al (as well as Poteat et al) study the authors aimed to replicate? This should either be reconsidered, or the value of the approach explicitly addressed in the manuscript.*

Thank you for this comment. As now explicated in the revised manuscript (lines 132-142), the rationale for our decision to include data presented in previous meta-analyses was: 1) Meta analyses have the potential to become more robust if more data is included. Instead of providing a disconnected pattern of meta-analytic summaries across a number of meta-analyses (with the danger of limited overlap), we chose to cover the whole period of studies available in order to provide a complete, comprehensive, and also nuanced understanding of the worldwide prevalence and burden of HIV among transgender individuals (line 134-136); 2) Because we presented two different approaches in our meta-analysis, it was imperative that we include the data that has been covered by previous meta-analyses. If those data were not included, there would be no evidence indicating if the differences found also hold based on data included in previous meta-analytic summaries.

*• While inclusion of both the pooled prevalence and standardized prevalence estimates by the authors is a laudable contribution to the field of transgender health research and HIV, the authors do not discuss the large discrepancies in the resulting estimates by method. A discussion of the results by method is warranted, particularly given the wide range in estimates produced by the two methods. Authors should provide context for why they believe the estimate vary to such a degree, and issues related to validity and reliability.*

Thank you for this comment. It is important to note that the standardized prevalence estimate was only applied for the overall prevalence estimation and we considered this to be methodologically more sound than a pooled prevalence estimate. In a pooled estimate, the total study population and total HIV cases are summed and then a crude proportion is calculated. This does not take heterogeneity and variation among the included studies into account. Our standardization approach entailed taking the weights from each country-year into account. Without the weighted standardization, a country-year combination that contains large study samples such as US-2010 (see Table 2; n=16,943 trans feminine individuals), or a country-year that includes small samples such as the Netherlands-2003 (Table 2; n=61 trans feminine individuals), may deliver misleading pooled results. The standardized prevalence by the weight from each country-year thus delivers a more robust estimation because it accounts for variations in study settings, sample sizes, and data quality. This argumentation is now included in the discussion section (lines 360-368).

*• A number of studies reporting laboratory confirmed HIV prevalence data for transgender populations that were captured in the previous reviews in this area detailed in the current manuscript seem to be missing from the current review. These include Rich et al. 2017 Culture, Health and Sexuality with data on transgender men, and several studies of transgender women.*

Thank you for your attention to detail here. We have gone back over previous reviews to ascertain if studies were missing. We established that five studies listed in those reviews were not included in our meta-analyses: 1) Bokhari et al., 2007; 2) Spizzichino et al., 2001; 3) Shrestha et al., 2011; 4) Simon et al., 2000; and 5) Rich et al., 2017. The first four were not included because they reported duplicate data. Their samplies overlapped with, respectively: 1) Khan et al., 2008; 2) Zacarelli et al., 2004; 3) Schulden et al., 2008; and 4) Reback et al., 2005. Their exclusion is in line with the procedure outlined in our methods section. To wit: "When studies reported duplicate data, the study with the smallest sample size was excluded." With respect to Rich et al., 2017, this was an oversight. Apologies. The study had a qualitative design and reported a prevalence rate of 0 with a sample of merely 11 participants. During data extraction, this was inadvertently allocated as an article not to be included. We have now corrected this. We have now included Rich et al., 2017 and rerun the analyses for trans masculine individuals. The manuscript has been adjusted to reflect this analyses and this can be seen in the abstract (lines 30-36) and the results section (lines 248-255, Table 3, and Figure 3). Overall, the conclusions are not impacted by the inclusion of the 11 additional participants in Rich et al., 2017.

*• The authors reference critiques of previously published reviews in this area, including the critique that the Baral et al. estimates may have been biased by inclusion of multiply marginalized samples of transgender women with heightened HIV risk factors in pooled prevalence estimation, citing this as a motivation for the sub-analysis in the current study by sampling method. However, this critique would be best addressed by sub-analysis by sample sub-population. An additional sub-analysis of studies captured in this review by sample sub-population (e.g. transgender sex workers, etc.) would be a major contribution to the literature. If not, a more robust discussion of the absence of such an analysis should be included in the limitations section.*

We appreciate the reviewer’s desire to optimize this manuscript. However, we believe that it is important that we collectively acknowledge the various demands that have been placed on this manuscript during the editorial process. Throughout this process, we have differentiated the analyses along a number of lines. Initially, this was along the lines of trans feminine and trans masculine populations, as well as geographical regions, sampling methods, and the introduction of PrEP. Later, we were asked to look at sampling year for each study, which we did. We were also asked to look by city, which was not feasible (see previous responses to reviewers and the limitations section). In this round, we have additionally been asked to differentiate analyses along the lines of sub-populations within the trans community (i.e., sex workers). Throughout the editorial process, we have accommodated the requests for which sufficient data is available on a global scale. While we acknowledge that it would be interesting to look at subgroups, the primary level data does not yet allow for all such analyses on a global scale. The subpopulation analysis now being requested would, similarly to the moderation by TasP analysis requested, be focused exclusively on US data which is not the global perspective we set out to take in this manuscript. With this in mind, we expanded the discussion section to include the following: “our analysis… did not separately ascertain prevalence rates for trans feminine individuals who engage in sex work versus those who do not as primary level data on this is not available on a global scale. We recommend that future research take these potential shortcomings into consideration. Specifically, we recommend that future research explicitly investigate prevalence among sub-populations within the transgender community… as this will provide an even more comprehensive picture of HIV prevalence and burden among transgender individuals.” (lines 435-442).
